# Titanium dioxide particles from the diet: involvement in the genesis of inflammatory bowel diseases and colorectal cancer

**DOI:** 10.1186/s12989-021-00421-2

**Published:** 2021-07-30

**Authors:** Frédérick Barreau, Céline Tisseyre, Sandrine Ménard, Audrey Ferrand, Marie Carriere

**Affiliations:** 1 INSERM, UMR 1220, Institut de Recherche en Santé Digestive, 31024 Toulouse, France; 2 Université de Toulouse, Toulouse, France; 3 Univ. Grenoble Grenoble Alpes, CEA, CNRS, IRIG-SyMMES, CIBEST, 17 rue des Martyrs, 38000 Grenoble, France

**Keywords:** TiO_2_, Food additive, Toxicity, Gastrointestinal tract, Ingestion, Intestine, Inflammation, Cancer

## Abstract

The gastrointestinal tract is a complex interface between the external environment and the immune system. Its ability to control uptake across the mucosa and to protect the body from damage of harmful substances from the lumen is defined as the intestinal barrier function (IBF). The IBF involves four elements: the intestinal microbiota, the mucus layer, the epithelium and the immune system. Its dysfunction is linked with human diseases including inflammatory, metabolic, infectious, autoimmune and neurologic disorders. Most of these diseases are complex and involve genetic, psychological and environmental factors. Over the past 10 years, many genetic polymorphisms predisposing to inflammatory bowel disease (IBD) have been identified. Yet, it is now clear that they are insufficient to explain the onset of these chronic diseases. Although it has been evidenced that some environmental factors such as cigarette smoking or carbohydrate intake are associated with IBD, other environmental factors also present potential health risks such as ingestion of food additives introduced in the human diet, including those composed of mineral particles, by altering the four elements of the intestinal barrier function. The aim of this review is to provide a critical opinion on the potential of TiO_2_ particles, especially when used as a food additive, to alter the four elements of the intestinal barrier function, and consequently to evaluate if this additive would likely play a role in the development and/or exacerbation of IBD.

**Supplementary Information:**

The online version contains supplementary material available at 10.1186/s12989-021-00421-2.

## Background

The gastrointestinal tract is a complex interface between the external environment, internal tissues and the immune system, establishing a dynamic barrier that enables the absorption of dietary nutrients and the exclusion of potentially hazardous substances, including pathogenic bacteria and toxic chemical agents, from the intestinal lumen. The ability to control their uptake across the mucosa and to protect from these harmful substances present within the lumen is defined as the intestinal barrier function (IBF)*.* It includes several lines of defence, the first one being the lumen itself, where commensal bacteria contribute to the protection against pathogens (i.e. harmful biological agents) by producing anti-microbial substances and by competing with them for access to nutrients, thereby limiting their colonisation [1]. The second line of defence is the mucus layer, which first constitutes a tight physical protection of the epithelium against all harmful substances that may transit in the gut lumen, and second is rich in secreted IgA and antimicrobial peptides (AMP), thereby preventing the access of bacteria to the epithelium [2]. The third barrier is the monolayer of intestinal epithelial cells that further refrains toxic molecules and enteric pathogens from reaching internal tissues [3]. This constitutes the last line of defence before the mucosal immune system, which constitutes the ultimate barrier against pathogens [1]. Many human diseases including inflammatory, metabolic, infectious, neurologic, and cardiovascular disorders are linked to deficiency of the IBF [4].

Crohn’s disease (CD) and ulcerative colitis (UC), the two main subtypes of inflammatory bowel diseases (IBD), are chronic relapsing inflammatory disorders of the gastrointestinal tract. CD can be distinguished from UC in that the inflammation associated with CD is transmural and often discontinuous [5]. IBD may be complicated by occlusions and perforations of the intestine. Patients suffering from IBD have a high risk of developing colitis-associated colorectal cancer (CAC) and have high mortality from these diseases [6, 7]. Indeed, chronic inflammation in the digestive tract results in particular pathological fibrosis that will affect the crypts. More importantly, in the majority of patients who did not show any signs of IBD pathogenesis prior to CRC onset, tumor-associated inflammation is evident in clinical samples and has been shown to drive cancer development in the gut, suggesting a fundamental role for inflammation in both CAC and sporadic colorectal cancer (CRC) development [8]. Current estimations indicate that 6.8 million people live with IBD worldwide [9]. The highest prevalence of IBD is in Europe and North America, but these countries show stabilized or decreased incidence of these diseases while their incidence is increasing in Africa, Asia, Eastern Europe and South America [10]. IBD is currently thought to be linked to environmental factors associated with the occidental way of life, which are currently unknown. Although cigarette smoking [11] or carbohydrate intake [12] are associated with CD, other environmental factors such as ingestion of food additives may present potential health risks by altering the IBF and then favouring IBD [13, 14]. Among them, the food additive E171, composed of titanium dioxide particles, has been shown to contain up to 55% of nanosized particles (TiO_2_-NPs) and is among the most commonly used mineral particle-based food additive in consumer products [15–18]. As reported in the present review, TiO_2_ has already been described to alter the IBF and to translocate in the internal body (for instance, see [19] where the absorption of TiO_2_ is demonstrated and [20, 21] where TiO_2_ is observed in the liver of rats exposed via oral administration). For this reason, this food additive is a good candidate as an environmental factor that could be implicated in IBD and CAC.

The aim of this review is to compile the published data that show how TiO_2_ food additive may impact the four elements of the IBF, in order to shed some light and to provide a critical opinion on the mechanisms by which it could be implicated in IBD and CAC. It is not intended to evaluate the risk associated with the ingestion of this additive, which has been already documented [22]. This review is based on a literature survey up to January 2021, using the databases Pubmed, IsiWeb and Scopus, which were searched with the keywords TiO_2_, titanium dioxide, E171, intestine, gut, inflammation, immunity, inflammatory bowel disease, mucus, microbiota, toxicity. The included publications were chosen thanks to quality criteria including minimal description of the origin and physico-chemical characteristics of the used TiO_2_ particles, good laboratory practices and correct description of the employed methods, particularly in the in vivo studies, conclusions matching the presented results and presence of appropriate controls.

## Description of the four compartments of the intestinal barrier function

### Commensal bacteria

The human gut contains around 10^14^ bacteria and constitutes the largest microbial community associated with human body [23]. The gut microbiota acts as a metabolic organ, through breakdown of indigestible dietary carbohydrates and proteins, with generation of fermentation end-products, vitamins, and ion absorption [24, 25]. It contributes to the barrier effect by constituting an obstacle to pathogen invasion. The microbiota also enhances the resistance to pathogens by both direct and indirect mechanisms of action including production of antimicrobial substances such as bacteriocins, pH modification and competition for nutrients, immune or epithelial cell-mediated mechanisms [1]. For example, *B. thetaiotaomicron* consumes carbohydrates used by *C. rodentium*, which contributes to the competitive exclusion of the pathogen. *B. thetaiotaomicron* also enhances the production by epithelial cells of the peptidoglycan-binding C-type lectin regenerating islet-derived protein IIIγ (REGIIIγ), which is an antimicrobial peptide that kills Gram^+^ bacteria [26].

### The mucus layer

The mucus layer overlying the epithelium promotes the elimination of gut content, providing the second line of defence against injury caused by ingested food, particles and microorganisms [27]. This layer is present all along the GIT, from the stomach to the rectum, and is thicker in the stomach and colon than in the small intestine [2]. It is classically divided into a dense sterile inner layer, which is firmly attached to the epithelium, and a removable outer layer that is colonized by anaerobic commensal bacteria [2]. In rodents, the mucus layer in the small intestine is not attached to the epithelium and only shows one layer [28]. The major components of the mucus layer are glycoproteins termed mucins, which are secreted by goblet cells and form a gel at the surface of epithelial cells [29]. In addition to mucins, goblet cells also synthesize trefoil factors, resistin-like molecule β, and Fc-γ binding protein [30]. Paneth cells also contribute to this barrier function by secreting substances contained in secretory granules, including antimicrobial substances such as lysozyme, phospholipase A2, α-defensins and the REGIIIγ [31]. Finally enterocytes produce β-defensins and REGIIIα [32, 33]. All these mucins and AMP compose the mucus wall and act synergistically to maintain the homeostasis of the mucus layer [29, 34].

### The epithelial monolayer

The epithelial monolayer is a semi-permeable barrier that allows the passage of water and nutrients from the gut lumen to the underlying tissues. Passage occurs via the paracellular or the transcellular route. The paracellular route, through tight junctions (TJs) that link adjacent cells, makes possible the strictly controlled passage of water, solutes and ions and is determined by the size of TJ pores with a maximum opening reaching 120 Å, i.e., 12 nm [35]. Four transmembrane families of proteins contribute to TJ formation, which are occludin, claudin, junctional adhesion molecule and tricellulin [35]. The intracellular domains of these transmembrane proteins interact with cytosolic scaffolding proteins, i.e. zonula occludens proteins, which in turn anchor the transmembrane proteins to the perijunctional actomyosin meshwork [35]. The circumferential contraction of the actinomyosin ring is regulated by the myosin light chain kinase [35, 36]. Other mechanisms like endocytosis of TJ proteins, regulation of TJ genes expression and epithelial cell apoptosis regulate the paracellular route. If TJs are not disrupted, they strictly regulate the passage of molecules and therefore the passage of nanoparticles with size > 120 Å, i.e., 12 nm, would be hindered.

A vast literature shows that particles larger than the TJ pore size would rather cross the gut epithelial layer by using the transcellular route, i.e. by passing through gut cells by transcytosis (for instance, see [36]). Although epithelial and dendritic cells are able to capture antigens and microbes from the gut lumen, full transcellular transport is mainly ascribed to microfold (M)-cells, which are part of the follicle associated epithelium (FAE) overlying isolated lymphoid follicles (ILF) or Peyer’s patches (PP) [37]. The transcellular route across M-cells and other cells exhibits two classical pathways depending on the size of the substance. Proteins and bacterial products can be taken up by endocytosis [38]. Larger entities, including bacteria, are captured via macropinocytosis or phagocytosis [38]. After crossing the cell, these particles are released on the other side of M-cells at the vicinity of immune cells that populate PPs. These two passage routes across the epithelium are closely controlled so that the intestinal homeostasis is maintained. In case of epithelial barrier disruption, enhanced passage of substances, including particles and microorganisms could lead to chronic inflammation [35].

### The intestinal immune system

This section gives a brief overview of the intestinal immune system, which is highly complex. Some excellent reviews describe its components and functioning, the authors suggest the reader to refer to the reviews cited in the text for more details. Two main sites of immune response have been described, i.e., the inductive site (immune response initiation – antigen encountering) and the effector site. Gut-associated lymphoid tissue (GALT), including PPs and ILFs, represent the inductive compartment for B and T cells. After encountering antigens in the GALT, B and T cells leave the effector site, transit through the lymph, enter the circulation and join the effector site of the intestine, that is, the mucosal epithelia and the lamina propria. Intestinal lymphocytes are continuously exposed to food and microbial antigens via antigen presenting cells (APC) including dendritic cells (DCs). DCs present in GALT and lamina propria have a high tolerogenic potential and a break of this tolerance triggers inflammation leading to IBD or cancer (for review, see [39–41]. These lymphocytes help to maintain the integrity of the intestinal barrier and immune homeostasis. Owing to their proximity to luminal antigens, they have a dual function: regulatory function (i.e., maintaining tolerance toward food antigens and commensal microbiota) and effector function (i.e., prevention of pathogenic invasion) (for review, see [42]). Innate lymphoid cells (ILCs) are also present in the lamina propria, those lymphocytes do not express the type of diversified antigen receptors expressed on T cells and B cells. ILCs are tissue-resident cells participating in tissue homeostasis (for review, see [43]). Intraepithelial lymphocytes (IELs) are also an important contributor to intestinal homeostasis. They reside within the intestinal epithelial layer and are involved in intestinal repair and defence against infections [44]. In IBD, alterations of these four elements constituting the IBF have been described.

## Absorption, distribution and elimination of TiO_2_ particles upon ingestion and associated toxicity

Upon request of the European Food Safety Authority (EFSA), a group of experts reviewed the nanomaterials that are currently used in agriculture, feed and food [45]. The food industry uses nanomaterials either in packaging or for nano-encapsulation of bioactive compounds such as vitamins. In addition, synthetic amorphous silica, titanium dioxide, silver and iron oxide are used as food additives for their anti-caking property or as food colorant [45]. Titanium dioxide food additive (E171) is a powder composed of particles with an average primary grain size higher than 100 nm. It also contains a significant amount of nanosized particles, which may arise from the production process. Depending on the analysed powder, this nanosized fraction is reported to be up to 36% [17], 35% [18], 55% [15] or 10–15% [16]. Thus, E171 is not a nanomaterial with respect to the current European recommendation for a definition of nanomaterials [46]. Its use in food products was authorized in 1969 by the JECFA (Joint FAE/WHO Expert Committee on Food Additives) with an acceptable daily intake “not specified” (report: NMRS 46/TRS 445-JECFA 13/12). This authorization relies on a literature survey that proved that E171 was non-toxic and not absorbed in the gastro-intestinal tract (GIT). In March 2021, the EFSA panel on Food Additives and Flavourings re-evaluated the safety of E171 when used as food additive, based on recent literature including a study dedicated to the analysis of E171 reproductive and developmental toxicity. The Panel concluded on potential immunotoxicity and inflammation with E171, as well as potential neurotoxicity and potential induction of aberrant crypt foci in the intestine. Moreover, a concern for genotoxicity could not be ruled out. Consequently, the Panel concluded that “E171 can no longer be considered as safe when used as a food additive” [47].

Since it is used as a white food colorant, the largest quantity of TiO_2_ is found in candies, sweets and pastries, with 0.01–1 mg TiO_2_ per unit [17]. In sugar-coated chewing gums, the nanoparticulate fraction of TiO_2_ reaches 27.7–43.7 and 95% of TiO_2_-NPs are swallowed upon chewing [48]. Based on consumer intake data, human exposure has been estimated at 1–2 mg and 0.2–0.7 mg TiO_2_/kg/day for US children under 10 and other US consumers, respectively [17]. More recent estimation concludes that daily intake of TiO_2_ for Dutch population is rather 0.66–0.70 mg/kg/day for children under 7, 0.16–0.18 mg/kg/day for consumers between 7 and 70, and 0.05–0.07 for adults over 70, with lifelong intake of TiO_2_ estimated at 0.19 mg/kg/day [49]. Lastly, the EFSA panel on food additives and nutrient sources estimates that daily intake of TiO_2_ reaches 0.4 mg/kg bw per day for infants and the elderly and 10.4 mg/kg bw per day for children, for the maximum level exposure assessment scenario, at the mean, which is the highest estimated values reported in their publication [50]. Based on the latter estimated values, and taking into consideration the existing guidance for conversion of doses between animals and humans such as the Nair and Jacob practice guide or the FDA’s guidelines, it can considered that a realistic in vivo exposure concentration would be up to 50–60 mg/kg b.w./day [51]. In vitro, exposing 2D-cells to 25 μg/cm^2^ (of cell culture surface) would represent ~ 10,000-fold the daily ingested dose by a human adult [52], therefore a realistic exposure concentration would be as low as 2.5 ng/cm^2^, i.e., ~ 5 ng/mL in classical 2D cell culture conditions. In such in vitro mechanistic studies, unrealistically high concentrations are often used in order to determine the effect and no-effect level of NMs [53]. Although it is recommended to use realistic concentrations and long-term exposure in vitro, such high concentrations can be considered as worst case scenarios and are acceptable as long as they are interpreted with caution and as they do not exceed the concentration at which NMs agglomerate or interfere with the readout of the test, i.e., generally 1–100 μg/mL [53].

Due to continuous exposure via ingestion, TiO_2_ might be distributed in intestinal tissues and in internal organs if absorbed through the gastrointestinal tract. Indeed, early reports describe the presence of so-called “pigmented cells” in human PPs. These cells contain some dark granules, which were analysed as being composed of Ti, Si and Al [54]. The authors propose that these pigments derive from the ingestion of synthetic food additive (Ti from TiO_2_), aluminosilicates (Al, Si) and mixed environmental silicates (Si). In addition to this local accumulation in immune cells, a recent study on human volunteers shows significant intestinal absorption of Ti after exposure via ingestion of two capsules containing 100 mg of food-grade TiO_2_ (which would represent 1–1.5 mg/kg b.w.) [19]. Ti is detected in the bloodstream of the volunteers as soon as 2 h after ingestion, and reflectance microscopy shows that Ti is in the physical form of a particle, not as ions that would have been released from the particles [19]. Moreover, the kinetics of appearance of Ti in the bloodstream suggests two routes of intestinal absorption, first in the proximal part of the small intestine, then in the distal part of the ileum, possibly through PPs [19]. In contrast, Jones et al. report insignificant absorption of TiO_2_ in human volunteers orally exposed to a single dose of 5 mg/kg b.w. of TiO_2_ micro and nanoparticles (15 nm, 100 nm and < 5 μm) dispersed in water [55]. However, in the Jones study the baseline level of Ti in the bloodstream is high, which impairs the detection of small amounts of Ti that would have been absorbed [55]. Finally, in 2017, Ruiz et al. show from a cohort study that the titanium level in the blood of human patients having active UC disease is higher than that of healthy donors [56].

Several in vivo studies in rodents report TiO_2_ absorption and toxicity when administered per os, although other studies report the opposite. When focusing on studies performed with E171 or TiO_2_ particles with physico-chemical properties that are close to those of E171, the literature is scarce. In 2015, three studies published in the same article clearly demonstrate absence of toxicity of TiO_2_ with physico-chemical characteristics close to those of food-grade TiO_2_. These three studies are performed according to the Organisation for Economic Co-operation and Development (OECD) guidelines for i) 90 day subchronic toxicity, ii) 28 days repeated dose toxicity and iii) acute oral toxicity [57]. In addition, negligible absorption, lack of distribution and an overall excretion in the feces is observed in rats exposed to E171 incorporated in food pellets at 30 mg/kg b.w./day for 7 days in a study conducted following the OECD TG417 [58]. Oral gavage of E171 at 10, 100 or 1000 mg/kg b.w./day for 90 days (OECD TG408) leads to accumulation of Ti only in the colon [59]. Conversely, some non-OECD guideline studies show the absorption and distribution of E171 in the liver of rats exposed for 7 days to E171 at 10 mg/kg b.w./day by intragastric gavage [20] and in mouse exposed by dripping some E171 into the mouth of the mouse at 5 mg/kg b.w./day, 3 days per week for 3 weeks [21].

In addition to this literature, some studies performed with TiO_2_ particles with properties differing from those of E171 report either no or significant absorption of TiO_2_ and in some of them some toxicity. Absorption of TiO_2_-NPs is reported for 25 or 80 nm NPs when administered to mice at a single dose of 5 g/kg b.w., which is an unrealistically high dose, with signs of toxicity in the liver, kidneys and brain as well as altered serum biochemical parameters [60]. The result of this study should be interpreted with caution due to the excessively high dose administered to the animals. Similarly, oral exposure of mice with 16 nm rutile or 20 nm anatase TiO_2_-NPs at the dose of 100 mg/kg/day for 28 days, which can also be considered as a high dose as compared to estimated human exposure, causes Ti distribution in the spleen, lung, and kidney without causing any significant effects on organ histology [61]. Jani et al. report in 1994 that 500 nm TiO_2_-NPs are absorbed in rats and accumulate in GALT, lungs and peritoneal tissues after oral administration at 12.5 mg TiO_2_/kg b.w. per day for 10 days. No Ti is detected in the liver and spleen [62]. More recently, a systematic study performed with five benchmark TiO_2_ particles from the JRC repository, differing in their physico-chemical characteristics, shows that none of these particles are significantly absorbed by rats after 5 consecutive days of oral exposure at the realistic concentration of 2.3 mg/animal per day (i.e., 7–12 mg/kg b.w. depending on the weight of the animal). No distribution to the liver, spleen and mesenteric lymph nodes is observed, except in a small number of animals [63]. This study also shows that when exposed by intravenous injection, TiO_2_ distributes mainly to the liver, spleen and lung. No TiO_2_ is detected in the urine and feces during the 90 days post-exposure, proving that its elimination is negligible [63]. This suggests that even if TiO_2_ absorption through the GI tract is mild, lifelong cumulative absorption combined to low elimination would result in potential accumulation of TiO_2_ in the tissues. Noteworthy, data from this study have been considered as the most accurate toxicokinetics data, and have been used for risk assessment of TiO_2_ oral exposure [22]. In contrast, while no TiO_2_-NP is detected in the liver, kidneys, spleen and brain of rats exposed by repeated gavage to 520, 1041 or 2083 mg/kg b.w./day for 13 weeks (OECD TG409) of 26 nm-diameter TiO_2_-NPs, TiO_2_ is significantly eliminated in the faeces [64]. The doses administered to rats in the latter study are unrealistic and higher than the dose in the study by Geraets et al. [63], which can explain the stronger elimination. Regarding TiO_2_ toxicity, while no significant distribution of TiO_2_ to the blood, liver, kidney and spleen is reported by Wang et al. in rats exposed to 10–200 mg/kg b.w. of 75 nm TiO_2_-NPs for 30 days, significant damage is observed both in young and adult animals [65]. In young rats, this repeated oral administration induces liver oedema and heart damage as well as cell activation in the stomach, but only at doses > 50 mg/kg b.w., which are higher than the estimated human exposure [65]. In adult rats, it causes damage to the liver, kidneys and compromises intestinal permeability, also at the highest doses, showing age-dependent impact of TiO_2_ [65]. Conversely, no systemic toxicity of P25 TiO_2_-NPs (21 nm in diameter, mix anatase/rutile) is observed in rats exposed for 28 or 90 days (OECD TG407 and 408) at 250, 500 or 1000 mg/kg b.w./day by gavage [66]. Finally, Ruiz et al. show dose-dependent distribution of TiO_2_ in the spleen of mice having dextran sodium sulfate (DSS)-induced colitis, when orally exposed to 30–50 nm rutile TiO_2_-NPs repeatedly for 7 days, at 50 or 500 mg/kg b.w./day [56]. This shows that the colitis caused by DSS facilitates absorption of TiO_2_ nanoparticles, which was expected as colitis perturbs intestinal permeability. Finally, a series of articles from the Fashui Hong team reports impact of TiO_2_ nanoparticles on diverse organs after oral administration, but these articles are not considered in this review because of flaws in the presentation and statistical analysis of the data, as has already been pointed out on a series of articles from the same team related to the pulmonary impact of similar TiO_2_ nanoparticles [67]. In these articles the reported standard errors are exactly 5% of the reported mean, rather than the actual standard errors.

Overall, it can be considered that TiO_2_ absorption is mild and its elimination in the faeces is slow, in realistic exposure conditions. Taking these conclusions into consideration, risk assessment for oral exposure to TiO_2_ concludes that a potential risk exists for liver, ovaries and testes [22]. Such an evaluation, including TiO_2_-NPs kinetics, indicates a possible effect on liver, spleen, ovaries and testes [22].

## Impact of TiO_2_ on the intestinal barrier function

Early reports suggest that ingestion of food additives composed of mineral particles such as TiO_2_ may participate in the aetiology and pathogenesis of IBD [13]. Current studies suggest that TiO_2_ particles alter all four elements of the IBF. Therefore, they may promote the development and/or the aggravation of IBD and the associated CRC.

### Alteration of the intestinal microbiota by TiO_2_ particles

The antibacterial properties of metals such as silver and zinc are known for centuries and this has been exploited in modern medicine for infection control. For example, silver nitrate was one of the first substances suggested for the management of dental cavities [68]. In addition, metal-containing nanomaterials arise in several chemical forms, including solid nanoparticles of metal or metal oxides like Ag or TiO_2_-NPs as well as composite materials with layers of distinct metals. Some of these nanoparticles are used as antibacterial agents [69] and there is now interest to use them for controlling bacterial infections [70]. The precise mechanisms for bacterial toxicity of these NPs is still being elucidated, but the current paradigms include free metal ion toxicity arising from the dissolution of metals from the surface of the NPs (e.g., Ag^+^ from Ag NPs) [71] or oxidative stress via the generation of reactive oxygen species (ROS) on the surface of some NPs.

It is unlikely that TiO_2_ nanoparticles, which are considered to be poorly soluble [72], can release sufficient quantities of Ti ion to confer them antibacterial properties. TiO_2_ is known as an antimicrobial agent because of its photocatalytic properties, which lead to the release of ROS when TiO_2_ is irradiated with UV light [73]. This property is currently used in many applications such as water treatment [74], but cannot explain the potential dysbiosis caused by TiO_2_ since no UV light reaches the GIT. Still, it is also demonstrated that TiO_2_ can generate some free radicals in the dark [75]. Such free radicals can then synergistically act by attacking polyunsaturated phospholipids in bacteria and damage their DNA [76, 77], which eventually may cause bacterial cell death. In the context of the GIT, such damage to bacteria could alter the intestinal microbiota.

Generally, the impact of TiO_2_ on the microbiota is reported to be mild (Table 1). Considering the E171 food additive, Radziwill-Bienkowska et al. show mild impact of E171 on a panel of Gram^+^/Gram^−^ bacterial strains chosen to be representative of gut microbiota [78]. E171 is internalized in 7% of *E. coli* cells, with minor consequences on bacterial viability [78]. According to Dudefroi et al., E171 does not affect bacterial gas production and only mildly impact bacterial fatty acid profiles in a defined human gut bacterial community omposed of 33 different strains (microbial ecosystem therapeutic 1, MET-1) [79]. DNA profiles and phylogenetic distributions confirm limited impact on the bacterial community, with a modest decrease of *Bacteroides ovatus* in favour to *Clostridium cocleatum* [79]. In a human colon model reactor, after 5 days of exposure at 36 mg/L (corresponding to oral exposure to ~ 0.3–0.7 mg/kg), both E171 and 21 nm TiO_2_-NPs hinder the natural shift in microbial composition that is observed in the control condition, favouring Firmicutes phylum over Proteobacteria [80]. The authors’ hypothesis is that this natural shift is probably due to the shift from a vegetarian diet (host) to a diet with high levels of animal proteins and fats (colon medium) [80]. After exposure to 21 nm TiO_2_-NPs, the amount of Firmicutes and Bacteroidetes increases, with Firmicutes being approximately as abundant as Proteobacteria [80]. Particles with diameter 122 nm, supposed to represent food grade TiO_2_ particles, trigger a slight decrease of Proteobacteria as well as a minor increase of Firmicutes and Bacteroidetes, but overall do not affect the initial ratio of these phyla. TiO_2_-NPs also decrease colonic pH, with 122 nm particles having a higher impact than 21 nm NPs [80]. The authors hypothesize that it could be due to a direct interaction between TiO_2_-NPs and bacteria, and/or binding of microbial nutrients at the particle surface [80]. More recently, a comprehensive study reports the impact of E171 or TiO_2_-NPs (33 nm in diameter) on the microbiota and the colon inflammatory status in non-obese and obese mice [81]. Exposure for 8 weeks to 0.1 wt% TiO_2_ incorporated into the feed leads to microbiota dysbiosis, characterized by increased abundance of Firmicutes and decreased abundance of Bacteroidetes phyla, i.e. the same trend as observed in IBD patients, together with reduced abundance of *Bifidobacterium* and *Lactobacillus* genera [81]. This dysbiosis concurs with decreased cecal levels of short-chain fatty acids (SCFAs), which has also been reported to be associated with IBD. Histological observation shows typical signs of inflammation in the colon, both in directly exposed animals and in control animals that have received fecal transplant from TiO_2_-exposed mice, suggesting direct impact of the microbiota on the intestinal inflammatory status [81]. Overall, these effects are more intense in obese mice compared to non-obese mice, and E171 shows mild impact while TiO_2_-NPs exert the strongest effect [81].

**Table 1:** Impact of TiO_2_ particles on the intestinal microbiota^a^

Study	Test system	Exposure conditions: dose range and exposure time	Material	Dispersion procedure	Main conclusions
in vitro					
Waller et al., 2016 [80]	Model human colon reactor: bacteria isolated from a fecal sample from a human donor, grown in aerobic condition	5 days, three times a days. TiO_2_ diluted in a fluid simulating digested food entering the large intestine. Exposure under flow, in the dark	P25 (Evonik, 21 nm, anatase/rutile) and commercially available TiO_2_, similar to food grade TiO_2_ (122 nm)	Sonication for 30 min in water	Decrease of bacteria number. Inhibition of the bacterial shift observed in control condition (Proteobacteria to Firmicutes phyla). Decreased colonic pH. Mild effect on microbial stability (microbial community hydrophobicity, electrophoretic mobility). Food-grade TiO_2_ particles show greater effect.
Dudefroi et al., 2017 [79]	MET-1 bacterial community (33 bacterial strains), grown in anaerobic condition	100–250 ppm. Incubation in the dark under agitation.	Two different E171 and NM105 TiO_2_-NP (21 nm, anatase/rutile)	Suspension in distilled water, no sonication	No impact on gas production by bacteria. Minor effect on fatty acids profiles. Limited effect on bacterial communities: decrease of *Bacteroides ovatus,* increase of *Clostridium cocleatum.*
Radziwill-Bienkowska et al., 2018 [78]	Eight Gram-positive/Gram-negative bacterial strains	32–320 μg/mL, 15 min to 24 h, in the dark, under agitation ot nor, depending on the strain.	E171 and NM105 TiO_2_-NP (22 nm, anatase/rutile)	Probe sonication in 0.05% water/BSA, 27 min	Adsorption of TiO_2_ on the surface of bacteria, with accumulation in 7% of *E. coli*, as measured by nano-SIMS. Alteration of growth profiles and moderate reduction of cell cultivability. Morphological damage on a small number of bacteria.
in vivo					
Chen et al., 2017 [82]	Mice, oral gavage	2,5 mg/kg b.w./day, once a day for 7 days	TiO_2_-NPs, anatase, 17 nm (TEM)	Suspension in water, bath sonication for 15 min	No change of gut microbiota composition.
Li et al., 2018 [61]	Mice, oral gavage	100 mg/kg b.w./day, once a day for 28 days	TiO_2_-NPs, anatase (20 nm, DLS) and rutile (16 nm, DLS)	Suspension in distilled water, no sonication	No decrease of gut microbiota diversity, but its structure is shifted: Proteobacteria increased by rutile TiO_2_, *Prevotella* decreased by anatase and rutile TiO_2_, *Rhodococcus* increased by rutile TiO_2_, *Bacteroides* increased by anatase TiO_2_.
Pinget et al., 2019 [133]	Mice, drinking water	2, 10, 50 mg TiO_2_/kg b.w./day for 21 days	E171	Suspension in drinking water	Limited impact on bacterial diversity, richness, evenness or Faith’s diversity in fecal samples in the colon. Impact at the genus level in the colon, depending on the dose. No impact in the small intestine. Significant modulation of commensal bacterial activity at 50 mg/kg b.w./day. Increased biofilm formation of E. coli, *E. faecalis* and commensal bacteria exposed to 2, 10 and 50 μg/mL for 24 h or 72 h, in vitro.
Cao et al., 2020 [81]	Mice, exposure in food pellets	0.1 wt% in food pellets for 8 weeks	E171 or TiO_2_-NPs, anatase, 33 nm (TEM)	Mixed with food, either low-fat or high-fat diet	Microbiota dysbiosis: increased abundance of Firmicutes and decreased abundance of Bacteroidetes; decreased abundance of *Bifidobacterium* and *Lactobacillus* genera, concurrent with decreased level of short-chain fatty acids. Associated with colon inflammation. More intense in obese mice and in mice treated with TiO_2_-NPs compared to E171. Microbiota transplant experiment suggests that inflammation is directly linked with dysbiosis.

^a^*Abbreviations*: *BSA* bovine serum albumin, *DLS* dynamic light scattering, *SIMS* secondary ion mass spectroscopy, *TEM* transmission electron microscopy

Other studies also report mild impact on the microbiota of TiO_2_ nanoparticles with physico-chemical properties that differ from E171. Chen et al. find no disturbance of gut microbiota in mice exposed by oral gavage to 19 nm TiO_2_-NPs, at 2.5 mg/kg/day for 7 days [82]. In faeces of both control and TiO_2_-NP exposed mice, the Bacteroidetes phylum is preponderant, followed by Firmicutes then Proteobacteria and exposure to TiO_2_-NPs does not affect their ratio [82]. Moreover, anatase or rutile TiO_2_-NPs, orally administrated to mice for 28 days at 100 mg/kg b.w./day, do not alter gut microbiota diversity but modifies its composition [61]. Rutile NPs have a more pronounced impact than anatase NPs [61]. The most altered phylum is Proteobacteria, which is significantly increased by rutile but not by anatase. At the genus level, the abundance of *Prevotella* is decreased by both TiO_2_-NPs, while the abundance of *Rhodococcus* is enriched by rutile NPs, and the abundance of *Bacteroides* is increased by anatase NPs [61]. Finally, food grade TiO_2_ minimally affects the composition of the microbiota in mice exposed in the drinking water, but it alters bacterial metabolite release and enhances biofilm formation from commensal bacteria, in vitro [83].

Overall, although some articles show that TiO_2_ may alter gut microbiota, other articles report the opposite. Therefore, this impact of TiO_2_ on gut microbiota is still a matter of debate. In vivo, human microbiome has been characterised, and its alleged composition varies among authors. However, Firmicutes and Bacteroidetes phyla are always shown to be predominant with a similar abundance, with Proteobacteria and Actinobacteria being far less present but still more abundant than all remaining phyla [84–86]. Dysbiosis has been linked to some intestinal pathologies, the most severe one being *Clostridium difficile* infection, but still alteration of the microbiota concurs with intestinal disorders such as IBD, irritable bowel disease and CRC [87]. For this reason any disturbance of the intestinal microbiota by TiO_2_ food additive could contribute to the aggravation of the symptoms of IBD and CRC in predisposed individuals.

### Impact on the epithelial monolayer

Studies assessing the impact of TiO_2_ on intestinal epithelial cells are summarized in Table 2. In vivo*,* exposure of mice to E171 at 50 mg/kg b.w./day for 4 weeks reduces the length of colonic crypts but not the total length of the colon [83]. Tight junction protein 1 (zonula occludens type 1, ZO-1) expression in the colon is not affected when mice are exposed to 10 or 50 mg/kg b.w./day of E171 for 4 weeks, which suggests no alteration of intestinal permeability [83]. Decreased crypt length is also observed in mice exposed for 16 weeks to 5 mg/kg b.w./day of E171, provided in the drinking water when the mice are fed with either a regular fat diet or a high-fat diet [88]. Conversely, Talamini et al. do not observe any structural and histological modifications in the intestine of mice exposed by dripping ~ 2 mg/kg b.w./day of E171 in the mouth of the mice, 3 days per week for 3 weeks [21]. The mode of administration of E171 could explain such apparently discrepant results.

**Table 2:** Impact of TiO_2_ particles on intestinal epithelium^a^

Study	Test system	Exposure conditions: dose range and exposure time	Material	Dispersion procedure	Main conclusions
Faust et al., 2014 [89]	Caco-2_BBe1_ cells, differentiated	Exposure using microgravity bioreactor (“floating” epithelia) for 24 h	E171 (anatase from XRD, 122 nm from TEM, “E171”) and E171 extracted from gum (141 nm, TEM, “gum E171”)	Probe sonication > 2 min, then dilution in exposure medium, then probe sonication > 2 min	Disruption of the brush border independently of particle sedimentation (in a microgravity reactor, or in inverted epithelium configuration): fewer microvilli, limp, less erected, at 350 ng/mL and 3.5 μg/mL of gum E171 or E171.
Brun et al., 2014 [52]	In vitro: differentiated Caco-2 and co-culture of Caco-2/HT29-MTX; ex vivo: Ussing chamber; in vivo: mice, oral gavage	In vitro: 50 μg/mL for 6 h, 24 h or 48 h; ex vivo: 50 μg/mL for 2 h; in vivo: 12.5 mg/kg b.w., sacrifice after 6 h	anatase (12 nm, TEM)	Probe sonication 30 min in water	In vitro: no impact on TEER value, paracellular and transcellular transport properties, activity of the P-gP. Upregulation of mRNA levels of proteins involved in tight and adherens junctions. In vivo and ex vivo: increased paracellular permeability after exposure to TiO_2_-NPs, downregulatio of mRNA expression of junction proteins (TJP1, TJP2, OCLN) in the ileum of mice exposed in vivo.
Dorier et al., 2015 [91]	Caco-2 cells, differentiated	50 μg/mL for 6 h, 24 h or 48 h	Anatase (12 nm, TEM) and rutile (20 nm, TEM)		Early and transient modulation of mRNA expression of ABC transporter genes, late modulation of mRNA expression of some SLC transporter genes. At 48 h significant increase of ABC transporter protein level (significant decrease at 4 h).
Guo et al., 2017 [92]	Co-culture of Caco-2/ HT29-MTX cells	10^6^, 10^8^ and 10^10^ particles/cm^2^ (low, medium, high concentration), for 4 h (acute) or 5 days (chronic)	TiO_2_-NPs, 20–40 nm from TEM	Suspension in water, mixing, no sonication	Chronic exposure decreased TEER value at the three doses (not acute), enlargement of the gaps between cells, as shown via OCLN protein immunostaining. Decreased Fe, Zn and fatty acid transport transport. mRNA expression of nutrient transporters affected.
Li et al., 2018 [61]	Mice (10 mice per group), oral gavage	100 mg/kg b.w./day, once a day for 28 days	Anatase (20 nm, DLS) and rutile (16 nm, DLS)	Suspension in distilled water, no sonication	Longer intestinal villi in the colon (mild effect) and disturbed arrangement of villus epithelial cells when exposed to rutile but not anatase.
Jensen et al., 2019 [134]	Rats, oral gavage once a week during 10 weeks	50 μg/kg b.w./week (low dose) and 500 μg/kg b.w./week (high dose)	E171 (anatase, sizes: 135 nm, 305 nm and 900 nm from TEM)	Indirect cup-type sonication (high energy) in 2% FBS, 16 min	Decreased mRNA expression of TJP1 in the colon mucosa at the high dose.
Dorier et al., 2019 [95]	Differentiated Caco-2 and co-culture of Caco-2/HT29-MTX	Repeated exposure (twice a week for 21 days); 10 and 50 μg/mL	E171 (anatase, 119 nm), A12 (anatase, 12 nm), NM105 (anatase/rutile, 21 nm)	Indirect cup-type sonication (high energy) in water, 30 min	No perturbation of the in vitro epithelial barrier development over the 21 days of exposure: TEER, mRNA expression of junction proteins and markers of microvilli differentiation unaffected. Decreased (E171) or decreased (NM105) levels of ABC transporters when repeated exposure.
Pinget et al., 2019 [133]	Mice, drinking water	2, 10, 50 mg TiO_2_/kg b.w./day for 21 days	E171	Suspension in drinking water	No impact on Tjp1 expression, suggesting no impact on intestinal permeability. Expression of Defb3, encoding beta-defensin 3, increased; granzyme B, cathelin-related antimicrobial peptide, regenerating islet-derived protein 3 gamma and p-lysozyme not significantly modulated. Reduction of colonic crypt length at 50 mg/kg b.w./day, no impact on colon length.
Talamini et al., 2019 [21]	Mice, TiO_2_ dripped into the mouse’s mouth	~ 2 mg/kg b.w./day 3 days/week for 3 weeks	E171	Suspension in water, no sonication, dripped into the mouth	No overt structural or morphological alteration in the stomach and intestine, no disruption of crypt structure, no atypical cell proliferation.
Medina-Reyes et al., 2020 [88]	Mice with either high fat diet (HFD) or regular diet (RD)	5 mg/kg b.w. for 16 weeks	E171	Suspension in drinking water	Decreased colon crypt length, as compared to the respective control (HFD or RD).
Zhang et al., 2021 [90]	Mice	1% w/w TiO_2_ for 1, 3 or 6 months		TiO_2_ incorporated in food pellets	Increased villi height/crypt depth ratios at 1 and 3 months; increased expression of ZO-1 and occludin at 1 month; spare microvilli in small intestine at 6 months. No change in intestinal permeability.

^a^*Abbreviations*: *ABC* ATP-binding cassette, *FBS* foetal bovine serum, *OCLN* occludin, *SLC* solute liquid carrier, *TEER* transepithelial resistance, *TEM* transmission electron microscopy, *TJP* tight junction protein, *XRD* X-ray diffraction

Although in vivo experiments are considered to be more informative than in vitro experiments on established cell lines, which are often derived from cancers, in vitro experiments might be useful to decrypt the mode of action of toxic substances such as nanomaterials. In vitro, E171 causes disruption of microvilli organization in Caco-2_BBe1_ cells exposed to concentrations of E171 as low as 100 ng/cm^2^ (i.e. 350 ng/mL) [89]. This disruption could be due to sedimentation of particles on the microvilli, which appears limp and fewer in number in exposed cells. When exposing Caco-2_BBe1_ cells to E171 using an inverted configuration, in which particles cannot sediment on top of the cell layer, disruption of microvilli is also observed [89]. Therefore, exposure to TiO_2_ affects the morphology of intestinal epithelium. Regarding the impact on intestinal epithelium of non-food-grade TiO_2_ nanoparticles, impact on intestinal villi and intestinal permeability is also reported. Li et al. show that daily gavage of mice for 28 days (100 mg/kg/day) with 16 nm rutile NPs but not with 21 nm anatase NPs results in longer intestinal villi and irregular arrangement of villus epithelial cells [61]. The ratio of villi height/crypt depth is modified in mice exposed for 1 or 3 months to anatase NPs or rutile microparticles via their feed (1% w./w.) and the level of ZO-1 and occludin is increased in the ileum after 1 month of exposure [90]. In vivo and ex vivo, agglomerates of 12 nm anatase TiO_2_-NPs alter the paracellular permeability of the ileum and colon epithelia of mice after a single oral gavage at 12.5 mg/kg b.w [52]. Gene expression of ZO-1 and -2, claudin 2 and 3 and occludin are down-regulated in the ileum, which is attributed by the authors to tight junction remodelling that may occur due to epithelial integrity alteration [52]. In the same study, in vitro exposure of a monoculture of Caco-2 cells (regular epithelium) and a co-culture of Caco-2 and HT29-MTX cells (mucus-secreting regular epithelium) to 50 μg/mL of these TiO_2_-NPs does not alter the paracellular permeability of the epithelia, nor transport through P-glycoprotein [52]. Moreover, acute exposure of Caco-2 cells to 50 μg/mL of anatase (12 nm) or rutile (20 nm) TiO_2_-NPs induces early upregulation of a battery of efflux pumps and nutrient transporters, suggesting that it might affect the efflux of toxic substances and nutrient absorption [91]. Moreover, another in vitro study demonstrates that, in addition to altering tight junctions, acute exposure for 4 h and repeated daily exposure for 5 days of Caco-2/HT29-MTX to 30 nm TiO_2_-NPs reduces absorptive cell microvilli, which are involved in nutrient absorption. This concurs with alteration of nutrient transport (specifically Fe and Zn) as well as fatty acid uptake [92]. The concentration used in this study are 10^6^, 10^8^ and 10^10^ particles/cm^2^ (acute exposure), or three times these concentrations (chronic exposure).

Overall, all these data highlight the fact that the protective and absorptive functions of intestinal epithelial cell can be impaired by TiO_2_ exposure.

### Impact of TiO_2_ on the mucus layer

Concerning the impact of TiO_2_ on the mucus and AMP secretions from epithelial cells including goblet cells, Paneth cells and enterocytes, the literature is scarce (Table 3). HT29-MTX cells, developed by Lesuffleur et al. [93], are an ideal model to study the interaction of NPs with the intestinal mucus, in vitro. The mucus is organized as islands on top of these cells, and these islands of mucus can trap E171 [94]. In vitro, repeated exposure of Caco-2/HT29-MTX cells for 21 days to 10, 50 or 100 μg/mL of E171 or 21 nm anatase/rutile TiO_2_-NPs causes mucus secretion to increase, while mucin gene expression remains unchanged [95]. In vivo, Medina-Reyes et al. show hyperplasia and hypertrophy of goblet cells and increased mucin gene expression in mice exposed for 16 weeks to 5 mg/kg b.w. of E171 in the drinking water and fed with either regular diet or high fat diet [88]. Inversely, intragastric administration of E171 to mice, both wild type and with chemically induced colitis-associated cancer, at 5 mg TiO_2_/kg b.w./day, 5 days a week during 10 weeks, decreases the number of goblet cells in the distal colon [96]. This would rather suggest that TiO_2_ reduces the capacity of mucus secretion in exposed animals. Moreover, Talbot et al. show no modification of cecal short-chain fatty acid (SCFA) profiles, which play a role in mucin synthesis and mucus characteristics, and of gut mucin O-glycosylation patterns in rats orally exposed to E171 or 21 nm anatase/rutile TiO_2_-NPs, which suggests that these particles show no significant effect on mucus quantity and quality [94]. In the latter study, rats have been exposed to 0.1 mg E171/kg b.w./day for 60 days or 10 mg E171/kg b.w./day for 7 days. While the concentration of E171 to which animals are exposed within the three latter studies are comparable and realistic, the exposure time varies from 7 days to 16 weeks. Their results point to the time-dependence of E171 impact on mucus secretion. They suggest that mucus secretion would not be affected at short exposure times (7 or 60 days) [94], it would then be reduced after 10 weeks of exposure [96] and finally increased after 16 weeks of exposure [88]. Still, such an interpretation should be treated with caution, as exposure patterns and E171 preparation procedure vary from one study to another. It is accepted that the procedure of TiO_2_ dispersion plays a pivotal role on its agglomeration state and biocorona; for instance TiO_2_-NPs agglomerate due to high ionic strength and when submitted to simulated digestion [97, 98], while E171 strongly adsorb some mucins on its surface, which leads to reduced agglomeration [99]. Thus, exposing animals via intragastric gavage or via drinking water would result in different biocorona on the particles and different agglomeration states, which could explain these apparently discrepant results.

**Table 3:** Impact of TiO_2_ particles on intestinal mucus^a^

Study	Test system	Exposure conditions: dose range and exposure time	Material	Dispersion procedure	Main conclusions
Urrutia-Ortega et al., 2016 [96]	Oral gavage, mice. Wild-type or model of CAC via AOM/DSS induction.	5 mg/kg body weight/5 days/10 weeks	E171	Sonication for 30 min in water	Reduced number of goblet cells in the distal colon, in both groups (WT and CAC), higher decrease in the CAC model.
Talbot et al., 2018 [94]	HT29-MTX and in vivo rat acute and sub-chronic oral exposure	In vitro: 250 μg/mL; in vivo acute (7 days, daily gavage): 10 mg/kg bw/day; in vivo sub-chronic (60 days, drinking water): 10 and 0,1 mg/kg bw/day	E171 and P25 (anatase/rutile, 21 nm)	Probe sonication in 0,05% water/BSA 27 min	Penetration of TiO_2_ into the mucoid area of HT29-MTX cells (patchy structures of mucus). When E171 and P25 were administered in vivo, cecal short-chain fatty acid profiles and gut mucin O-glycosylation patterns remained unchanged
Dorier et al., 2019 [95]	Caco-2/HT29-MTX co-culture	Acute (6-48 h; 50 μg/mL) or repeated twice a week for 3 weeks; 10 and 50 μg/mL)	E171, anatase 12 nm (A12), anatase/rutile, 21 nm (P25)	Indirect cup-type sonication (high energy) 30 min in water	No impact on cell differentiation. Inflammatory profile (chronic and acute), increased mucus secretion (chronic). Epithelial integrity unaltered, content of ABC family of xenobiotic efflux pumps modified (either increased or decreased depending on the particle: decreased with E171, increased with P25).
Pinget et al., 2019 [133]	Mice, drinking water	2, 10, 50 mg TiO_2_/kg b.w./day for 21 days	E171	Suspension in drinking water	Decreased *Muc2* gene expression in the colon at 10 and mg/kg b.w./day
Medina-Reyes et al., 2020 [88]	Mice with either high fat diet (HFD) or regular diet (RD)	5 mg/kg b.w. for 16 weeks	E171	Suspension in drinking water	Hyperplasia and hypertrophy of goblet cells; increased Muc2, Muc5AC, Muc1 and Muc13 mRNA expression.

^a^*Abbreviations*: *ABC* ATP-binding cassette, *AOM* azoxymethane, *BSA* bovine serum albumin, *CAC* model of colitis-associated cancer, *DSS* dextran sulfate sodium, *WT* wild-type

To put into perspective these recent studies, an abundant literature demonstrates the impact of TiO_2_-NPs on the mucus secretion from the respiratory system and on the development of the pulmonary diseases like asthma, chronic obstructive pulmonary disease or cystic fibrosis. Analysis of this literature is reported as Supplementary materials and suggests the implication of calcium signalling in the induction of mucus secretion triggered by TiO_2_ [100].

### Impact on the intestinal immune system

A paradigm of TiO_2_ toxicity, especially via inhalation, is that it causes inflammation [101]. Several studies have also reported that TiO_2_ would induce an inflammatory profile in the intestine when ingested (Table 4). In rats orally exposed for 7 days to 10 mg/kg b.w./day of E171, increased DC (CD103 + MHCII+) population and decreased helper T cells (Th) (CD4 + CD25+) and regulatory T cells (Treg) (CD4 + CD25 + Foxp3+) populations were observed in PPs [20]. This observation of immune cells population shift is completed by functional in vitro experiments through TcR stimulation of isolated cells from PP and spleen. Interestingly, E171 decreases CD3/CD28-induced IFNγ at the intestinal level but increases its secretion at the systemic level. Higher IL-17 secretion is also observed in the spleen of rats exposed to E171 without modification at the intestinal level [20]. Moreover, 100 days of oral exposure to E171 at 10 mg/kg b.w./day lead to decrease of Th and Treg populations in PP [20]. This inflammatory signature could lead to IBD in susceptible individuals. In contrast, Blevins and al. do not report any modification of DCs, nor Th, nor Treg in PPs after 7 or 100 days of exposure to E171 at 400 mg/kg of bw/day, when E171 is incorporated in the feed [102]. However, after 100 days of E171 oral exposure, IL-17 concentration is higher in the colon of exposed animals [102]. So far, the consequences of oral exposure to TiO_2_ on intestinal ILC, IEL or B cells populations are unknown. Accumulation of TiO_2_-NPs inside the intestinal cells, especially in PP, might lead to damaging outcomes such as inflammation and has been postulated as being potentially involved in the pathogenesis of IBD [13, 62, 103]. In 2012, Nogueira et al. evidenced that TiO_2_-NPs induce a pro-inflammatory response in the small intestine by increasing inflammatory cytokine production and T CD4+ cell proliferation [104]. TiO_2_ induces recruitment of CD4+ T cells in the ileum, as shown via histological analyses, associated with higher secretion of IL-12, IFNγ, TNFα, IL-4, IL-23 and TGF-β [104], which suggests that TiO_2_ commits immune response toward Th1. Moreover, hypertrophy and hyperplasia of the small intestinal mucosa are also observed in mice exposed to TiO_2_-NPs. This induction of inflammation in the small intestine is probably linked to the presence of more M-cells in this area (PP and ILF), which are known to be the main pathway of nanoparticle uptake across the gastrointestinal tract [105, 106]. Although there remains some ambiguity surrounding the specific immunomodulatory and cytotoxic effects resulting from in vivo TiO_2_-NPs exposure, it seems clear that TiO_2_ has the potential to cause immune cell and tissue damage and dysfunction.

**Table 4:** TiO_2_ and intestinal pathologies^a^

Study	Test system	Exposure conditions: dose range and exposure time	Material	Dispersion procedure	Main conclusions
*Inflammatory bowel disease*					
Powell et al., 1996 [54]	Human GALT from donors with CD, UC, and colon carcinoma	n/a	n/a	n/a	Presence of particles in phagolysosomes of macophages from GALT (“pigmented” cells). Anatase TiO_2_ (100–200 nm), flakes of aluminosilicates with some Fe on the surface (< 100–400 nm), mixed silicates without aluminium (100–700 nm).
Powell et al., 2000 [123]	Human intestinal biopsies from donors with CD, UC and healthy donors, EL4.NOB-1 murine lymphoma lymphoblasts, CTLL murine T cells	5 μg/mL TiO_2_ or 5 μg/mL TiO_2_, together with 1 ng/mL LPS and 4 mM CaCl_2_ or 1 ng/mL LPS	Anatase TiO_2_, 0.2 μm	Suspension in culture medium, or suspension together with LPS and CaCl_2_ (ion bridge) followed by ultrasonication	Increased secretion of IL-1 in cells and organ cultures stimulated with the mixture of TiO_2_ and LPS, not with TiO_2_ or LPS alone.
Nogueira et al., 2012 [104]	Mice	Oral gavage, 100 mg/kg bw/day; 10 days	TiO_2_-NPs (66 nm, home-made) and MP-TiO_2_ (260 nm, Kronos 1171, food-grade)	Suspension in distilled water, sonicated (no indication of the method) immediately before administration	Increased levels of CD4+ T cells in duodenum, jejunum and ileum of animals exposed to TiO_2_. Increased levels of IL-12, IL-4, IL-23, TNF-α, IFN-γ and TGF-β (pro-inflammatory), suggesting a Th1-mediated inflammatory response. Hypertrophy and hyperplasia of the mucosal epithelium in the duodenum, jejunum and ileum. Effects of NPs and MPs are not significantly different.
Ammendolia et al., 2014 [126]	*Listeria monocytogenes* (LM2 and LM9) biofilms and Caco-2 cells	0.8, 8 and 80 μg/mL TiO_2_ for 24 h and 48 h	commercial TiO_2_-NPs, anatase, 139 nm mean diameter	Probe sonication in bacterial medium for 45 min	Exposure of bacteria to TiO_2_-NPs, then exposure of Caco-2 cells to TiO_2_-exposed bacteria. No impact on bacterial cell survival. Increased biofilm production for LM2 with 0.8, 8 and 80 μg/mL TiO_2_ for 24 h and for LM9 with 80 μg/mL TiO_2_ for 24 or 48 h. Modification of the biofilm structure. Increased interaction of LM2 with Caco-2 cells when exposed to TiO_2_, reduced intracellular accumulation of LM2 and LM9, reduced multiplication of LM2 and LM9 in Caco-2 cells when exposed to TiO_2_.
Hummel et al., 2014 [124]	Biopsies from children suspected to having IBD	n/a	n/a	n/a	Amount of pigment (defined in Powell et al., 2000 as agglomerates of mineral particles) in Peyer’s patches. The amount of pigment increased with the age of patients. Significantly less pigment in patients supposed to having CD as compared to healthy donors and patients suspected to having UC.
Urrutia-Ortega et al., 2016 [96]	Mice, or mice with chemically-induced CAC (12.5 mg/kg body weight/ip AOM and 2% DSS)	Oral gavage, 5 mg/kg b.w./day, 5 days a week, 10 weeks	E171	Suspension in water, sonication for 30 min at 60 Hz. 300 nm in diameter (DLS)	Transient inflammation observed in the CAC group, more intense in the CAC + E171 group. Decreased interleukin expression (IL-2, TNF-α, INF-γ, IL-10) in the CAC + E171 group, compared to the E171 group. Migration of TNF-α from the cytoplasm to the nucleus of colorectal epithelial cells in E171 and CAC groups.
Ruiz et al., 2016 [56]	Mice, wild-type or deficient in (NLRP)3, with chemically-induced colitis (DSS). In vitro: Caco-2, HT29 and other epithelial cells, THP-1 treated or not with siRNA for caspase-1, ASC and NLRP3. Bone marrow-derived macrophages (from mice). Human patients with IBD having active disease	In vivo: oral gavage, 50 or 500 mg TiO_2_/day/kg b.w. for 7 days (WT) or 500 mg TiO_2_/day/kg b.w. for 7 days (Nlrp3−/− mice). In vitro: exposure to 5, 20 or 100 μg/mL of TiO_2_ for 24 h.	In vivo: 30–50 nm, rutile. In vitro: TiO_2_ rutile (30–50 nm) and anatase with size ca 0.36 μm (food-grade)	Suspension in drinking water (in vivo); suspension in ultrapure water and sonication for 5 min (in vitro)	In mice: no sign of colitis in WT mice exposed to TiO_2_ with no pre-existing colitis. In mice with chemically-induced colitis: colitis symptoms are worsened in WT mice, not in Nlrp3−/− mice. In vitro: both TiO_2_ particles triggers NLRP3-ASC-caspase-1 assembly, caspase-1 cleavage and the release of NLRP3-associated IL-1β and IL-18. TiO_2_ induces intracellular ROS accumulation. In human volunteers with IBD: Higher TiO_2_ level in the bloodstream compared to healthy patients.
Bettini et al., 2017 [20]	Rats or rats with chemically-induced carcinogenesis (DMH)	Oral gavage 10 mg/kg of b.w./day, 7 days; or exposure via the drinking water at 200 μg or 10 mg/kg of b.w./day for 100 days	E171 and NM105 (21 nm, anatase/rutile TiO_2_-NP)	Probe sonication for 16 min in water/BSA 0.05%	Disturbance in intestinal and systemic immune homeostasis, colon microinflammation after 100 days of treatment.
Chen et al., 2017 [82]	Mice	Oral gavage, 2.5 mg/kg b.w./day, once a day for 7 days	TiO_2_-NPs, anatase (not food-grade)	suspension in water, sonication in a water bath at 12–15 °C for 15 min	No evidence of inflammation (only a few inflammatory cells observed), no pathological changes by H&E staining. Increase of IL-1β in the colon only (measurement of IL-1β, IL-6 and TNF-α in the colon and small intestine).
Hong et al., 2017 [135]	Mice	Oral gavage, 1.25, 2.5, and 5 mg/kg b.w./day, once a day for 9 months	TiO_2_-NPs, anatase (not food-grade), 5–6 nm	Suspension in 0.5% w/v HPMC, ultrasonication for 45 min and mechanic vibration for 10 min	Inflammatory cell infiltration in the stomach. Increased protein expression of NF-κB, TNF-α, IL-lβ, IL-6, IL-8, COX-2, and PGE2 in the stomach, decreased protein expression of IκB.
Dorier et al., 2019 [95]	Caco-2/HT29-MTX co-culture	Acute (6-48 h; 50 μg/mL) or repeated twice a week for 3 weeks; 10 and 50 μg/mL)	E171, A12 (inhouse synthesis, anatase, 12 nm), P25 (anatase/rutile, 21 nm)	Suspension in distilled water, sonication indirect cup-type sonicator in continuous sonication mode for 30 min at 52.8 W (80% amplitude)	No impact on cell differentiation. Inflammatory profile (chronic and acute), increased mucus secretion (chronic). Epithelial integrity unaltered, content of ABC family of xenobiotic efflux pumps modified (either increased or decreased depending on the particle: decreased with E171, increased with P25).
Pinget et al., 2019 [83]	mice, drinking water	2, 10, 50 mg TiO_2_/kg b.w./day for 21 days	E171	Suspension in drinking water	Proliferation of macrophages in the colonic myeloid compartment of mice exposed at 10 or 50 mg/kg b.w./day, no impact on the population of neutrophiles, dendritic cells, total monocytes. Upregulation of IL-6, TNF-α and IL-10 in the colon.
Blevins et al., 2019 [102]	Rats, exposure to TiO_2_ included in food pellets	40, 400 or 5000 pm in food pellets, 7 or 100 days	E171	n/a	No effect on immune parameters: percentage of dendritic, CD4+ T or Tregs in Peyer’s patches, in their periphery. No effect on cytokine release in the colon, jejunum and plasma.
*cancer*					
Urrutia-Ortega et al., 2016 [96]	Mice, or mice with chemically-induced CAC (12.5 mg/kg body weight/ip AOM and 2% DSS)	Oral gavage 5 mg/kg bw/5 days a week/10 weeks	E171	1 mg/ml in water, water and sonication for 30 min at 60 Hz	E171 in non-chemically-induced animals: no tumor formation but in the distal colon: slight vascularization, increase of crypts size and number, hyperplastic epithelium with slight dysplastic changes. CAC: purulent material and blood discharge; abnormal behavior; well differentiated adenocarcinomas in the distal region of the colon. E171 in CAC: exacerbated blood and purulent discharge and higher inflammation with rectal prolapse (vs CTL CAC animals) + abnormal behavior. Enhanced the tumor formation in distal colon; mildly differentiated adenocarcinoma. Increased fluorescence of markers of tumor progression. Decrease IL2, IL10, TNFα, INFγ. Kidney weight reduced. Both groups: decrease of goblet cells in the distal colon, higher in CAC + E171.
Bettini et al., 2017 [20]	Rats or rats with chemically-induced carcinogenesis (DMH)	Oral gavage 10 mg/kg of BW/day, 7 days; or exposure via the drinking water at 200 μg or 10 mg/kg of BW/day for 100 days	E171	Probe sonication for 16 min in water/BSA 0.05%	Increased number of aberrant cryt foci per colon after 100 days of treatment with E171 at 10 mg/kg b.w./day, proving initiation of preneoplastic lesions. In DMH-induced rats, increased number of aberrant crypts per colon and of large aberrant crypt foci per colon after exposure for 100 days at 10 mg E171/kg b.w./day, proving promotion of carcinogenesis.
Proquin et al., 2017 [129]	Mice	Oral gavage, 5 mg/kg b.w./day, 2, 7, 14 and 21 days (5 times per week)	E171	1 mg/ml in water, water and sonication for 30 min at 60 Hz	Analysis of the colon. Structure of crypts altered, hyperplastic epithelium. No effect on intestinal cell proliferation. Transcriptome analysis with pathways affected: olfactory/GPCR receptor family, cancer, oxidative stress, cell cycle, immune response, metabolism of proteins.
Proquin et al., 2018 [130]	Mice with chemically-induced CAC (12.5 mg/kg body weight/ip AOM and 2% DSS)	Oral gavage, 5 mg/kg b.w./day, 2, 7, 14 and 21 days (5 times per week)	E171	1 mg/ml in water, water and sonication for 30 min at 60 Hz	Analysis of the colon. Transcriptome analysis with pathways affected: olfactory/GPCR receptor family, cancer, metabolism of xenobiotics, immune system, oxidative stress.
Setyawati et al., 2018 [132]	SW480 cells, in vitro	100, 250 and 500 μM, 4 days	TiO_2_-NP, 21 nm (P25 Evonik)	Sonication (no detail) in 0.5% FBS DMEM	Epithelial to mesenchymal (EMT) transition: phenotypic change (fibroblastic elongated morphology, lost of cell-cell junctions, diffuse actin staining), modulation of levels of EMT marker proteins: alpha-SMA, VIM, E-cadherin. Cell mobility and invasive behavior increased. Decreased cell proliferation. Activation of the TGF-beta (not via Smad3, rather via MAPK) pathway, concommittant with ROS level elevation. Activation of the Wnt pahway.
Blevins et al., 2019 [102]	Rats, exposure to TiO_2_ included in food pellets	40, 400 or 5000 pm in food pellets, 7 or 100 days	E171	n/a	No effect on histopathological observation: aberrant crypt foci, goblet cell number, colonic gland length.
Talamini et al., 2019 [21]	Mice, TiO_2_ dripped into the mouse’s mouth	~ 2 mg/kg b.w./day 3 days/week for 3 weeks	E171	Suspension in water, no sonication, dripped into the mouth	No atypical cell proliferation in the intestine.
Medina-Reyes et al., 2020 [88]	Mice with either high fat diet (HFD) or regular diet (RD)	5 mg/kg b.w. for 16 weeks	E171	Suspension in drinking water	Increased number of adenomas in the colon. No difference in the volume of adenomas. No abnormal cell proliferation (as probed via the PCNA and Ki67 proliferation markers mRNA expression)

^a^*Abbreviations*: *AOM* azoxymethane, *ABC* ATP-binding cassette, *BSA* bovine serum albumin, *CAC* colitis-associated cancer, *CD* Crohn’s disease, *CTL* control, *DLS* dynamic light scattering, *DMH* 1,2-dimethylhydrazine, *DSS* dextran sulfate sodium, *GALT* gut-associated lymphoid tissue, *H&E* hematoxylin and eosin, *HPMC* hydroxypropyl methyl cellulose, *IBD* inflammatory bowel disease, *LPS* lipopolysaccharide, *PMA* phorbol 12-myristate 13-acetate, *UC* ulcerative colitis

To our knowledge, only these three recent articles report in vivo consequences of TiO_2_ exposure –only two of them having been conducted with food-grade TiO_2_- on the intestinal immune system. The difficulty to characterize intestinal immune response could explain this lack of data. Therefore, to complete this section, we include some data related to the consequences of oral exposure to TiO_2_-NPs with characteristics differing from those of E171 on the systemic immune system, i.e., in the spleen, liver, PBMC and microglia. In vivo, administration of TiO_2_-NPs has been demonstrated to have multiple immunomodulatory effects, characterized by TiO_2_-NPs distribution in peripheral lymphoid organs, alterations of immune cell number, viability and function. Although they do not recapitulate the complexity of the local intestinal immune system, simple in vitro or ex vivo models using systemic immune cells could inform on the mechanisms of TiO_2_-driven immune cell dysfunctions. TiO_2_-NPs (5–6 nm) administered by intragastric gavage at 2.5, 5 or 10 mg/kg b.w./day for 90 days cause macrophage infiltration in the spleen [107]. Moreover, mice gavage with 2.5, 5 or 10 mg/kg b.w./day of the same 5–6 nm anatase TiO_2_-NPs for six consecutive months causes distribution of TiO_2_-NPs in the liver, associated with liver dysfunction, infiltration of inflammatory cells and altered mRNA levels of Th-1 and Th-2 type cytokines, i.e., IL-4, IL-5 and IL-12, as well as their targets IFN-γ, GATA3, GATA4, T-bet, STAt3, STAT6, eotaxin, MCP-1, and MIP-2 [108]. In vitro, treatment with TiO_2_-NPs of the peripheral tissue equivalent (PTE) or the modular immune in vitro construct (MIMIC) system, which is an immunological construct comprising blood vein endothelial cells (HUVECs) and peripheral blood mononuclear cells (PBMCs), also shows some inflammatory response. Indeed, treatment with TiO_2_-NPs leads to a dose-dependent elevation of inflammatory cytokines including IL-1α, IL-1β, IL-6, IL-8, IL-10, IFN-γ and TNF-α, as well as upregulated expression of the maturation marker CD83 and the co-stimulatory molecule CD86 on dendritic cells [109]. Notably, 48 h of exposure to TiO_2_-NPs also results in a 10–20-fold increase of ROS levels in PBMCs [109]. Treatment of RAW 264.7 macrophages with very low concentrations of TiO_2_-NPs, such as those that might be found in consumer products, induces NF-κB activation, which results in upregulation of pro-inflammatory gene expression [110]. Similarly, TiO_2_-NPs alter the expression of adhesion molecules and the chemokine receptor type 4 (CXCR4) in PBMCs and trigger an increase in T cell proliferation in response to PHA stimulation at concentrations that are not cytotoxic to the immune cells [111]. The direct exposure of microglia (which is also a tissue specific macrophage-like cell) to TiO_2_-NPs elevates the iNOS expression associated to an increase of NO release from the microglia [112]. The expression levels of MCP-1, MIP-1α, TNFα, IL-1β and IL-6 are also upregulated, as is the activation of NF-κB [112]. Further demonstrating the direct impact of TiO_2_-NPs on immune cells, TiO_2_-NPs induce the expression of NOD-like receptor family, pyrin domain containing 3 (NLRP3) inflammasome by dendritic cells and macrophages [113, 114]. TiO_2_-NPs activate NLRP3 inflammasome in macrophages via a mechanism that is dependent upon the release of ATP. Namely, the activation of phospholipase C-β has been shown to be necessary for TiO_2_-NP induced NLRP3 activation and for the resulting IL-1β production. IL-1β production in response to TiO_2_-NPs is augmented by ATP and ADP hydrolysis, and suppressed by adenosine degradation or selective A2_A_ or A2_B_ receptor inhibition [115].

Although multiple studies indicate that TiO_2_-NPs induce immune cell activation and the subsequent release of inflammatory factors, TiO_2_-NP exposure also possesses the potential to cause immune cell death. For example, exposure of dendritic cells to TiO_2_-NPs induces a dose-dependent decrease of cell viability, with concentrations of TiO_2_ nanoparticles in the range of 10–100 μM eliciting an approximate 20% decline in viability [109]. Human dermal fibroblasts capable of exhibiting inflammatory responses also show a time and dose-dependent decrease in cell viability upon exposure to TiO_2_ nanoparticles, however, an increase in ROS levels and expression of several pro-inflammatory genes including the TLRs, genes of the MAP kinase cascade and IL-1β is observed. Notably, several mediators of the NF-κB pathway are activated: ERK1/2 expression is upregulated and p38 is phosphorylated. Interestingly, NF-κB knockdown fibroblasts are resistant to TiO_2_ nanoparticle-induced cell death, suggesting that the activation of the NF-κB pathway plays a role in TiO_2_ nanoparticle-induced immune cell death [116]. In conclusion, although they are not strictly derived from experiments involving the intestinal immune system and the E171 food additive, these data underscore the ability of TiO_2_-NPs to modulate multiple aspects of immune function.

## Potential implications of TiO_2_ particles in chronic intestinal pathologies

Despite much research on this topic, the pathophysiology of IBD and associated CRC are not well understood. The IBD pathogenesis model is based on the dysregulation of the normally controlled immune response to commensal bacteria, which could be precipitated by infection or by defects in the mucosal barrier. This involves infiltration of immune cells producing chemokines and cytokines, which in turn exacerbate the dysfunctional immune response and activate either Th1, Th2 or Th17 cells in the gut mucosa of IBD patients [117]. More precisely, the pathogenesis of IBD occurs via three distinct stages: (i) penetration of luminal contents into underlying tissues which may be facilitated by environmental factors like infection [38], toxic particles or inherent defects in mucosal barrier linked with genetic mutations [118, 119], (ii) impaired clearance of luminal material from the bowel wall [120, 121] and (iii) a compensatory acquired immune response which leads to a chronic inflammatory response and gives rise to characteristic IBD lesions and further on CRC [122].

### Implication of TiO_2_ particles in inflammatory bowel diseases

Various specific and non-specific environmental factors have been associated with the induction and/or exacerbation of disease activity in patients with CD and UC [54, 123]. Among them, some papers have suspected that TiO_2_ from food may have a detrimental role in the pathogenesis of the IBD (Table 4). The first study to evidence a link between particles and alterations of the immune cell of the GALT is the article of Powell et al., published in 1996 [54]. It shows the presence of microparticles, including TiO_2_ particles, in the phagolysosomes of macrophages populating the GALT and suggests that the pathogenicity of these microparticles should be further investigated. Indeed, in susceptible individuals, the same intracellular distribution of these three types of particles can cause chronic latent granulomatous inflammation which is one cardinal feature of CD [54]. The same group of researchers has also evidenced that ultrafine particles of TiO_2_ strongly enhance the secretion of inflammatory cytokines by peripheral blood mononuclear cells and intestinal macrophages from IBD patients [123]. This result demonstrates that dietary particles are not immunologically inert and may be important adjuncts in overcoming normal gut cell hypo-responsiveness to endogenous luminal molecules [123]. As already discussed in the previous sections of this review, the same type of microparticles is observed in children having IBD or not; 42% of the patients show these pigmented cells, with significant difference between healthy children, children with UC and children with CD [124]. Patients with CD show lower number of pigment cells as compared with non-IBD patients and patients with UC [124]. A hypothesis to explain this observation is that these pigmented cells are involved in the inflammatory process in CD patients, and consequently they migrate from their initial site of activation to active sites of the inflammatory response [124]. Therefore, these microparticles may play a role in the inflammatory process of CD [124].

Since these discoveries, some researches have focused on the impacts of these fine and ultrafine particles on IBD development and/or exacerbation. As described in the previous sections of this manuscript, TiO_2_ may impact the gut microbiota. In addition to the perturbations described in this previous sections, recent studies evidence that TiO_2_-NPs enhance the development of the infection and/or the immune cells response to LPS. Due to its non-degradability, TiO_2_ can also interact with intestinal microorganisms or their products and induce or aggravate inflammatory processes. For example, bacterial lipopolysaccharide (LPS), abundant in the gut, avidly binds TiO_2_-NPs surface, facilitated by calcium-bridging cations and mucosal secretions. This complex induces release of inflammatory cytokines in primary human mononuclear phagocytes [125] or in intestinal explants [123]. Similarly, other studies evidence that 139 nm TiO_2_-NP exposure, at 80 μg/mL, influences both production and structural architecture of the biofilm synthetized by *Listeria monocytogenes* [126] and that food-grade TiO_2_ promotes biofilm formation of *E. coli, E. faecalis* and mice commensal bacteria, in vitro [83]. Moreover, TiO_2_-NPs alter bacterial interaction with the intestinal cells [126]. Thus, increased biofilm production due to TiO_2_ exposure may favour bacterial survival in the environment and its transmission to animal and human hosts. In addition to its role on the gut microbes, including bacteria (pathogenic or not) and viruses, as developed in the previous sections of this review, TiO_2_-NPs are also able to disrupt the integrity of three others components of the intestinal mucosa: epithelium, mucus layer and the immune system. TiO_2_-NPs also have a synergistic effect with some bacterial product to aggravate the inflammation [127]. As TiO_2_-NPs can aggravate intestinal inflammation, it is of interest to investigate their pathogenicity on models mimicking IBD patients, such as in a study by Ruiz et al., which shows that oral administration of TiO_2_ NPs triggered the aggravation of acute colitis in a dextran sodium sulfate (DSS) mouse model of colitis through the activation of the NLRP3 inflammasome [56]. As already commented, these TiO_2_ NPs accumulate in a dose-dependent manner in the spleen of these mice, highlighting the fact that ingested TiO_2_ NPs can cross an altered intestinal barrier and reach the systemic circulation. Those results were corroborated by the fact that, after oral administration of TiO_2_ NPs, patients with active ulcerative colitis presented increased levels of titanium in their systemic circulation [56].

### Implication of TiO_2_ particles in colorectal cancer

The International Agency for Research on Cancer (IARC), after comprehensively studying its hazardous potential, has rated TiO_2_ as possibly carcinogenic for humans by inhalation (Group 2B) [128]. This classification, in addition to the fact that TiO_2_ can disrupt the intestinal barrier and induce intestinal inflammation, calls for studies investigating the impact of titanium on intestinal carcinogenesis. While TiO_2_ does not appear as carcinogenic for healthy mice, it enhances the formation of intestinal tumours in mice suffering from induced colitis associated cancer (CAC) [96] (Table 4). This aggravated tumour formation is linked to dysplastic changes in the colonic epithelium, but also to a dramatic decrease in goblet cells (mucin-secreting cells) leading to intestinal barrier disruption [96]. Intestinal cancer formation could also be favoured by gene expression alterations caused by TiO_2_, and in this perspective transcriptomic studies show that food-grade TiO_2_ affects the response to oxidative stress, the immune response, DNA repair and carcinogenesis [129, 130]. Tumorigenesis could also be linked to ROS production as well as to DNA damage triggered by the interaction of E171 with microtubules or oxidative DNA damage in cells acutely or repeatedly exposed to E171 or TiO_2_-NPs [15, 131]. In addition, TiO_2_-NPs have been reported to promote the epithelial to mesenchymal transition (EMT) process in colorectal cancer cells [132]. More precisely, TiO_2_-NPs activate the transforming growth factor-β (TGF-β)/mitogen-activated protein kinase (MAPK) and wingless (Wnt) pathways that drives the EMT process [132]. Moreover, the colon of rats exposed for 100 days to E171 presents pre-neoplastic lesions, this exposure condition favouring the development of aberrant crypt foci in a chemically induced carcinogenesis model [20]. Nevertheless, when provided to rats in a food matrix, E171 do not produce such effects [102], suggesting that the food matrix would be protective for the intestinal epithelium. Taken together, although some studies support the fact that exposure to TiO_2_ could promote the development of CRC, additional studies must be conducted to validate or exclude a deleterious impact of these particles on colonic stem cell capacity to derive in carcinogenic cells.

## Conclusions and perspectives

In conclusion, these data indicate that TiO_2_ is able to alter the four compartments of IBF and to induce a low-grade intestinal inflammation associated or not with pre-neoplastic lesions. However, since multiple evidence has shown that the intestinal homeostasis in adults results directly from the perinatal establishment of the IBF, it is of major importance to investigate the impact of TiO_2_ for the early phases of the intestinal development, i.e., during the embryogenic and lactating periods. Moreover, since IBD is a complex disease involving genetic, psychologic and environmental factors, it is important to check the impact of this material in models exhibiting IBD susceptibility like models mutated in the genes encoding the *NOD2*, *ATG16L1*, *IRGM* or *IL-23* receptors. Finally, to demonstrate the likely involvement of these particles in the IBD genesis, it is also crucial to investigate whether exposure to these particles in combination with episodes of stress, which are described to be involved in the IBD, may induce an intestinal inflammation and linked colorectal cancer similar to those observed in IBD, in mice with genetic susceptibility to IBD.

## Supplementary Information

**Additional file 1.**

## Data Availability

Not applicable.

## Abbreviations

TiO_2_: Titanium dioxide
IBD: Inflammatory bowel disease
AMP: Antimicrobial peptide
CAC: Colitis-associated cancer
CD: Crohn’s disease
CXCR4: Chemokine receptor type 4
EFSA: European food safety agency
FAE: Follicle-associated epithelium
GALT: Gut-associated lymphoid tissue
IBF: Intestinal barrier function
ILF: Isolated lymphoid follicle
JECFA: Joint FAE/WHO expert committee on food additives
MAPK: Mitogen-activated protein kinase
NRLP3: NOD-like receptor family, pyrin domain containing 3
OECD: Organisation for economic co-coperation and development
PBMC: Peripheral blood mononuclear cell
PP: Peyer’s patches
REGIIIγ: Regenerating islet-derived protein 3
ROS: Reactive oxygen species
TGFβ: Transforming growth factor β
Th: T-helper
IARC: International agency for research on cancer
TJ: Tight junction
SAS: Synthetic amorphous silica
UC: Ulcerative colitis
Wnt: Wingless and Int-1

## References

[1] Perez-Lopez A, Behnsen J, Nuccio SP, Raffatellu M (2016). Mucosal immunity to pathogenic intestinal bacteria. Nat Rev Immunol.

[2] McGuckin MA, Lindén SK, Sutton P, Florin TH (2011). Mucin dynamics and enteric pathogens. Nat Rev Microbiol.

[3] Peterson LW, Artis D (2014). Intestinal epithelial cells: regulators of barrier function and immune homeostasis. Nat Rev Immunol.

[4] König J, Wells J, Cani PD, García-Ródenas CL, MacDonald T, Mercenier A (2016). Human Intestinal Barrier Function in Health and Disease. Clin Transl Gastroenterol.

[5] Podolsky DK (2002). Inflammatory bowel disease. N Engl J Med.

[6] Feagins LA, Souza RF, Spechler SJ (2009). Carcinogenesis in IBD: potential targets for the prevention of colorectal cancer. Nat Rev Gastroenterol hepatol.

[7] Lakatos PL, Lakatos L (2008). Ulcerative proctitis: a review of pharmacotherapy and management. Expert Opin Pharmacother.

[8] Grivennikov SI, Karin M (2010). Inflammation and oncogenesis: a vicious connection. Curr Opin Genet Dev.

[9] Collaborators GIBD (2020). The global, regional, and national burden of inflammatory bowel disease in 195 countries and territories, 1990-2017: a systematic analysis for the global burden of disease study 2017. Lancet Gastroenterol Hepatol.

[10] Ng SC, Shi HY, Hamidi N, Underwood FE, Tang W, Benchimol EI, Panaccione R, Ghosh S, Wu JCY, Chan FKL, Sung JJY, Kaplan GG (2017). Worldwide incidence and prevalence of inflammatory bowel disease in the 21st century: a systematic review of population-based studies. Lancet.

[11] Cosnes J, Carbonnel F, Carrat F, Beaugerie L, Cattan S, Gendre J (1999). Effects of current and former cigarette smoking on the clinical course of Crohn's disease. Aliment Pharmacol Ther.

[12] Chan SS, Luben R, van Schaik F, Oldenburg B, Bueno-de-Mesquita HB, Hallmans G (2014). Carbohydrate intake in the etiology of Crohn's disease and ulcerative colitis. Inflamm Bowel Dis.

[13] Lomer MC, Thompson RP, Powell JJ (2002). Fine and ultrafine particles of the diet: influence on the mucosal immune response and association with Crohn's disease. Proc Nutr Soc.

[14] Rogler G, Vavricka S (2015). Exposome in IBD: recent insights in environmental factors that influence the onset and course of IBD. Inflamm Bowel Dis.

[15] Dorier M, Beal D, Marie-Desvergne C, Dubosson M, Barreau F, Houdeau E (2017). Continuous in vitro exposure of intestinal epithelial cells to E171 food additive causes oxidative stress, inducing oxidation of DNA bases but no endoplasmic reticulum stress. Nanotoxicology.

[16] Peters RJ, van Bemmel G, Herrera-Rivera Z, Helsper HP, Marvin HJ, Weigel S (2014). Characterization of titanium dioxide nanoparticles in food products: analytical methods to define nanoparticles. J Agric Food Chem.

[17] Weir A, Westerhoff P, Fabricius L, Hristovski K, von Goetz N (2012). Titanium dioxide nanoparticles in food and personal care products. Env Sci Technol.

[18] Yang Y, Doudrick K, Bi X, Hristovski K, Herckes P, Westerhoff P, Kaegi R (2014). Characterization of food-grade titanium dioxide: the presence of nanosized particles. Env Sci Technol.

[19] Pele LC, Thoree V, Bruggraber SF, Koller D, Thompson RP, Lomer MC (2015). Pharmaceutical/food grade titanium dioxide particles are absorbed into the bloodstream of human volunteers. Part Fibre toxicol.

[20] Bettini S, Boutet-Robinet E, Cartier C, Comera C, Gaultier E, Dupuy J (2017). Food-grade TiO2 impairs intestinal and systemic immune homeostasis, initiates preneoplastic lesions and promotes aberrant crypt development in the rat colon. Sci Rep.

[21] Talamini L, Gimondi S, Violatto MB, Fiordaliso F, Pedica F, Tran NL, Sitia G, Aureli F, Raggi A, Nelissen I, Cubadda F, Bigini P, Diomede L (2019). Repeated administration of the food additive E171 to mice results in accumulation in intestine and liver and promotes an inflammatory status. Nanotoxicology.

[22] Heringa MB, Geraets L, van Eijkeren JC, Vandebriel RJ, de Jong WH, Oomen AG (2016). Risk assessment of titanium dioxide nanoparticles via oral exposure, including toxicokinetic considerations. Nanotoxicology.

[23] Eckburg PB, Bik EM, Bernstein CN, Purdom E, Dethlefsen L, Sargent M, Gill SR, Nelson KE, Relman DA (2005). Diversity of the human intestinal microbial flora. Science.

[24] Falony G, Vlachou A, Verbrugghe K, De Vuyst L (2006). Cross-feeding between Bifidobacterium longum BB536 and acetate-converting, butyrate-producing colon bacteria during growth on oligofructose. Appl Environ Microbiol.

[25] Macfarlane S, Macfarlane GT (2003). Regulation of short-chain fatty acid production. Proc Nutr Soc.

[26] Vaishnava S, Yamamoto M, Severson KM, Ruhn KA, Yu X, Koren O, Ley R, Wakeland EK, Hooper LV (2011). The antibacterial lectin RegIIIgamma promotes the spatial segregation of microbiota and host in the intestine. Science.

[27] Mayer L (2003). Mucosal immunity. Pediatrics..

[28] Ermund A, Schütte A, Johansson ME, Gustafsson JK, Hansson GC (2013). Studies of mucus in mouse stomach, small intestine, and colon. I. Gastrointestinal mucus layers have different properties depending on location as well as over the Peyer's patches. Am J Physiol Gastrointest Liver Physiol.

[29] Hollingsworth MA, Swanson BJ (2004). Mucins in cancer: protection and control of the cell surface. Nat Rev Cancer.

[30] Kim YS, Ho SB (2010). Intestinal goblet cells and mucins in health and disease: recent insights and progress. Curr Gastroenterol Rep.

[31] Antoni L, Nuding S, Wehkamp J, Stange EF (2014). Intestinal barrier in inflammatory bowel disease. World J Gastroenterol.

[32] Natividad JM, Hayes CL, Motta JP, Jury J, Galipeau HJ, Philip V (2014). Differential induction of antimicrobial REGIII by the intestinal microbiota and Bifidobacterium breve NCC2950. Appl Environ Microbiol.

[33] Wehkamp J, Harder J, Weichenthal M, Mueller O, Herrlinger KR, Fellermann K, Schroeder JM, Stange EF (2003). Inducible and constitutive beta-defensins are differentially expressed in Crohn's disease and ulcerative colitis. Inflamm Bowel Dis.

[34] Makkink MK, Schwerbrock NM, Mahler M, Boshuizen JA, Renes IB, Cornberg M (2002). Fate of goblet cells in experimental colitis. Dig Dis Sci.

[35] Turner JR (2009). Intestinal mucosal barrier function in health and disease. Nat Rev Immunol.

[36] Keita AV, Soderholm JD (2010). The intestinal barrier and its regulation by neuroimmune factors. Neurogastroenterol Motil.

[37] Keita AV, Soderholm JD (2012). Barrier dysfunction and bacterial uptake in the follicle-associated epithelium of ileal Crohn's disease. Ann N Y Acad Sci.

[38] Barreau F, Hugot J (2014). Intestinal barrier dysfunction triggered by invasive bacteria. Curr Opin Microbiol.

[39] Gaudino SJ, Kumar P (2019). Cross-talk between antigen presenting cells and T cells impacts intestinal homeostasis, bacterial infections, and tumorigenesis. Front Immunol.

[40] Sun T, Nguyen A, Gommerman JL (2020). Dendritic cell subsets in intestinal immunity and inflammation. J Immunol.

[41] Yang ZJ, Wang BY, Wang TT, Wang FF, Guo YX, Hua RX, Shang HW, Lu X, Xu JD (2021). Functions of dendritic cells and its association with intestinal diseases. Cells.

[42] Ma H, Tao W, Zhu S (2019). T lymphocytes in the intestinal mucosa: defense and tolerance. Cell Mol Immunol.

[43] Rankin L, Groom J, Mielke LA, Seillet C, Belz GT (2013). Diversity, function, and transcriptional regulation of gut innate lymphocytes. Front Immunol.

[44] Ribot JC, Lopes N, Silva-Santos B (2021). γδ T cells in tissue physiology and surveillance. Nat Rev Immunol.

[45] Peters RJB, Bouwmeester H, Gottardo S, Amenta V, Arena M, Brandhoff P, Marvin HJP, Mech A, Moniz FB, Pesudo LQ, Rauscher H, Schoonjans R, Undas AK, Vettori MV, Weigel S, Aschberger K (2016). Nanomaterials for products and application in agriculture, feed and food. Trends Food Sci Technol.

[46] Commission Recommendation of 18 October 2011 on the definition of nanomaterial (Text with EEA relevance) (2011/696/EU). Official Journal of the European Union. 2011.

[47] Younes M, Aquilina G, Castle L, Engel KH, Fowler P, Frutos Fernandez MJ (2021). Safety assessment of titanium dioxide (E171) as a food additive. EFSA Journal.

[48] Chen XX, Cheng B, Yang YX, Cao A, Liu JH, Du LJ (2013). Characterization and preliminary toxicity assay of nano-titanium dioxide additive in sugar-coated chewing gum. Small.

[49] Rompelberg C, Heringa MB, van Donkersgoed G, Drijvers J, Roos A, Westenbrink S, Peters R, van Bemmel G, Brand W, Oomen AG (2016). Oral intake of added titanium dioxide and its nanofraction from food products, food supplements and toothpaste by the Dutch population. Nanotoxicology.

[50] EFSA Panel on Food Additives and Nutrient Sources added to Food (ANS) (2016). Re-evaluation of titanium dioxide (E 171) as a food additive. EFSA Journal.

[51] Hashem MM, Abo-El-Sooud K, Abd-Elhakim YM, Badr YA, El-Metwally AE, Bahy-El-Dien A (2020). The long-term oral exposure to titanium dioxide impaired immune functions and triggered cytotoxic and genotoxic impacts in rats. J Trace Elem Med Biol.

[52] Brun E, Barreau F, Veronesi G, Fayard B, Sorieul S, Chaneac C (2014). Titanium dioxide nanoparticle impact and translocation through ex vivo, in vivo and in vitro gut epithelia. Part Fibre Toxicol.

[53] Drasler B, Sayre P, Steinhauser KG, Petri-Fink A, Rothen-Rutishauser B (2017). In vitro approaches to assess the hazard of nanomaterials. Nanoimpact.

[54] Powell JJ, Ainley CC, Harvey RS, Mason IM, Kendall MD, Sankey EA, Dhillon AP, Thompson RP (1996). Characterisation of inorganic microparticles in pigment cells of human gut associated lymphoid tissue. Gut.

[55] Jones K, Morton J, Smith I, Jurkschat K, Harding AH, Evans G (2015). Human in vivo and in vitro studies on gastrointestinal absorption of titanium dioxide nanoparticles. Toxicol Let.

[56] Ruiz PA, Moron B, Becker HM, Lang S, Atrott K, Spalinger MR (2017). Titanium dioxide nanoparticles exacerbate DSS-induced colitis: role of the NLRP3 inflammasome. Gut.

[57] Warheit DB, Brown SC, Donner EM (2015). Acute and subchronic oral toxicity studies in rats with nanoscale and pigment grade titanium dioxide particles. Food Chemical Toxicol.

[58] Farrell TP, Magnuson B (2017). Absorption, distribution and excretion of four forms of titanium dioxide pigment in the rat. J Food Sci.

[59] Han HY, Yang MJ, Yoon C, Lee GH, Kim DW, Kim TW, Kwak M, Heo MB, Lee TG, Kim S, Oh JH, Lim HJ, Oh I, Yoon S, Park EJ (2020). Toxicity of orally administered food-grade titanium dioxide nanoparticles. J Appl Toxicol.

[60] Wang J, Zhou G, Chen C, Yu H, Wang T, Ma Y, Jia G, Gao Y, Li B, Sun J, Li Y, Jiao F, Zhao Y, Chai Z (2007). Acute toxicity and biodistribution of different sized titanium dioxide particles in mice after oral administration. Toxicol Lett.

[61] Li J, Yang S, Lei R, Gu W, Qin Y, Ma S, Chen K, Chang Y, Bai X, Xia S, Wu C, Xing G (2018). Oral administration of rutile and anatase TiO2 nanoparticles shifts mouse gut microbiota structure. Nanoscale.

[62] Jani PUMD, Florence AT (1994). Titanium dioxide rutile particle uptake from the rat GI tract and translocation to systemic organs after oral-administration. Int J Pharma.

[63] Geraets L, Oomen AG, Krystek P, Jacobsen NR, Wallin H, Laurentie M, Verharen HW, Brandon EFA, de Jong WH (2014). Tissue distribution and elimination after oral and intravenous administration of different titanium dioxide nanoparticles in rats. Part Fibre Toxicol.

[64] Cho WS, Kang BC, Lee JK, Jeong J, Che JH, Seok SH (2013). Comparative absorption, distribution, and excretion of titanium dioxide and zinc oxide nanoparticles after repeated oral administration. Part Fibre Toxicol.

[65] Wang Y, Chen Z, Ba T, Pu J, Chen T, Song Y, Gu Y, Qian Q, Xu Y, Xiang K, Wang H, Jia G (2013). Susceptibility of young and adult rats to the oral toxicity of titanium dioxide nanoparticles. Small.

[66] Heo MB, Kwak M, An KS, Kim HJ, Ryu HY, Lee SM (2020). Oral toxicity of titanium dioxide P25 at repeated dose 28-day and 90-day in rats. Part Fibre Toxicol.

[67] Møller P, Wallin H, Cassee FR, Loft S (2018). Does intranasal instillation TiO(2) cause pulmonary tumorigenesis in male mice?. Environ Toxicol.

[68] Hill TJAF (1937). The effect of silver nitrate in the prevention of dental caries. J Dent Res.

[69] Li Q, Mahendra S, Lyon DY, Brunet L, Liga MV, Li D, Alvarez PJJ (2008). Antimicrobial nanomaterials for water disinfection and microbial control: potential applications and implications. Water Res.

[70] Allaker RP (2010). The use of nanoparticles to control oral biofilm formation. J Dent Res.

[71] Kim JS, Kuk E, Yu KN, Kim JH, Park SJ, Lee HJ, Kim SH, Park YK, Park YH, Hwang CY, Kim YK, Lee YS, Jeong DH, Cho MH (2007). Antimicrobial effects of silver nanoparticles. Nanomedicine.

[72] Sohal IS, Cho YK, O'Fallon KS, Gaines P, Demokritou P, Bello D (2018). Dissolution behavior and biodurability of ingested engineered nanomaterials in the gastrointestinal environment. ACS Nano.

[73] Schneider J, Matsuoka M, Takeuchi M, Zhang J, Horiuchi Y, Anpo M, Bahnemann DW (2014). Understanding TiO2 photocatalysis: mechanisms and materials. Chem Rev.

[74] Laxma Reddy PV, Kavitha B, Kumar Reddy PA, Kim KH (2017). TiO(2)-based photocatalytic disinfection of microbes in aqueous media: a review. Environ Res.

[75] Fenoglio I, Greco G, Livraghi S, Fubini B (2009). Non-UV-induced radical reactions at the surface of TiO2 nanoparticles that may trigger toxic responses. Chemistry.

[76] Wong MS, Chu WC, Sun DS, Huang HS, Chen JH, Tsai PJ (2006). Visible-light-induced bactericidal activity of a nitrogen-doped titanium photocatalyst against human pathogens. Appl Environ Microbiol.

[77] Hirakawa K, Mori M, Yoshida M, Oikawa S, Kawanishi S (2004). Photo-irradiated titanium dioxide catalyzes site specific DNA damage via generation of hydrogen peroxide. Free Radic Res.

[78] Radziwill-Bienkowska JM, Talbot P, Kamphuis JBJ, Robert V, Cartier C, Fourquaux I, Lentzen E, Audinot JN, Jamme F, Réfrégiers M, Bardowski JK, Langella P, Kowalczyk M, Houdeau E, Thomas M, Mercier-Bonin M (2018). Toxicity of food-grade TiO2 to commensal intestinal and transient food-borne Bacteria: new insights using Nano-SIMS and synchrotron UV fluorescence imaging. Front Microbiol.

[79] Dudefoi W, Moniz K, Allen-Vercoe E, Ropers MH, Walker VK (2017). Impact of food grade and nano-TiO2 particles on a human intestinal community. Food Chem Toxicol.

[80] Waller TCC, Walker SL (2017). Food and Industrial Grade Titanium Dioxide Impacts Gut Microbiota. Environ Eng Sci.

[81] Cao X, Han Y, Gu M, Du H, Song M, Zhu X (2020). Foodborne Titanium Dioxide Nanoparticles Induce Stronger Adverse Effects in Obese Mice than Non-Obese Mice: Gut Microbiota Dysbiosis, Colonic Inflammation, and Proteome Alterations. Small.

[82] Chen HZR, Wang B, Cai C, Zheng L, Wang H, Wang M, Ouyang H, Zhou X, Chai Z, Zhao Y, Feng W (2017). The effects of orally administered ag, TiO2 and SiO2 nanoparticles on gut microbiota composition and colitis induction in mice. NanoImpact.

[83] Lamas B, Martins Breyner N, Houdeau E (2020). Impacts of foodborne inorganic nanoparticles on the gut microbiota-immune axis: potential consequences for host health. Part Fibre Toxicol.

[84] Arumugam M, Raes J, Pelletier E, Le Paslier D, Yamada T, Mende DR (2011). Enterotypes of the human gut microbiome. Nature.

[85] Das B, Ghosh TS, Kedia S, Rampal R, Saxena S, Bag S (2018). Analysis of the Gut Microbiome of Rural and Urban Healthy Indians Living in Sea Level and High Altitude Areas. Sci Rep.

[86] Human Microbiome Project C (2012). Structure, function and diversity of the healthy human microbiome. Nature.

[87] Tojo R, Suárez A, Clemente MG, de los Reyes-Gavilán CG, Margolles A, Gueimonde M (2014). Intestinal microbiota in health and disease: role of bifidobacteria in gut homeostasis. World J Gastroenterol.

[88] Medina-Reyes EI, Delgado-Buenrostro NL, Díaz-Urbina D, Rodríguez-Ibarra C, Déciga-Alcaraz A, González MI, Reyes JL, Villamar-Duque TE, Flores-Sánchez MLO, Hernández-Pando R, Mancilla-Díaz JM, Chirino YI, Pedraza-Chaverri J (2020). Food-grade titanium dioxide (E171) induces anxiety, adenomas in colon and goblet cells hyperplasia in a regular diet model and microvesicular steatosis in a high fat diet model. Food Chem Toxicol.

[89] Faust JJ, Doudrick K, Yang Y, Westerhoff P, Capco DG (2014). Food grade titanium dioxide disrupts intestinal brush border microvilli in vitro independent of sedimentation. Cell Biol Toxicol.

[90] Zhang Y, Duan S, Liu Y, Wang Y (2021). The combined effect of food additive titanium dioxide and lipopolysaccharide on mouse intestinal barrier function after chronic exposure of titanium dioxide-contained feedstuffs. Part Fibre Toxicol.

[91] Dorier M, Brun E, Veronesi G, Barreau F, Pernet-Gallay K, Desvergne C, Rabilloud T, Carapito C, Herlin-Boime N, Carrière M (2015). Impact of anatase and rutile titanium dioxide nanoparticles on uptake carriers and efflux pumps in Caco-2 gut epithelial cells. Nanoscale.

[92] Guo Z, Martucci NJ, Moreno-Olivas F, Tako E, Mahler GJ (2017). Titanium dioxide nanoparticle ingestion alters nutrient absorption in an in vitro model of the small intestine. NanoImpact.

[93] Lesuffleur T, Porchet N, Aubert JP, Swallow D, Gum JR, Kim YS, Real FX, Zweibaum A (1993). Differential expression of the human mucin genes MUC1 to MUC5 in relation to growth and differentiation of different mucus-secreting HT-29 cell subpopulations. J Cell Sci.

[94] Talbot P, Radziwill-Bienkowska JM, Kamphuis JBJ, Steenkeste K, Bettini S, Robert V (2018). Food-grade TiO2 is trapped by intestinal mucus in vitro but does not impair mucin O-glycosylation and short-chain fatty acid synthesis in vivo: implications for gut barrier protection. J Nanobiotechnol.

[95] Dorier M, Beal D, Tisseyre C, Marie-Desvergne C, Dubosson M, Barreau F (2019). The food additive E171 and titanium dioxide nanoparticles indirectly alter the homeostasis of human intestinal epithelial cells in vitro. Environ-Sci Nano.

[96] Urrutia-Ortega IM, Garduno-Balderas LG, Delgado-Buenrostro NL, Freyre-Fonseca V, Flores-Flores JO, Gonzalez-Robles A (2016). Food-grade titanium dioxide exposure exacerbates tumor formation in colitis associated cancer model. Food Chem Toxicol.

[97] Cao XQ, Zhang T, DeLoid GM, Gaffrey MJ, Weitz KK, Thrall BD (2020). Evaluation of the cytotoxic and cellular proteome impacts of food-grade TiO2 (E171) using simulated gastrointestinal digestions and a tri-culture small intestinal epithelial model. Nanoimpact.

[98] Marucco A, Prono M, Beal D, Alasonati E, Fisicaro P, Bergamaschi E, Carriere M, Fenoglio I (2020). Biotransformation of food-grade and Nanometric TiO(2) in the Oral-gastro-intestinal tract: driving forces and effect on the toxicity toward intestinal epithelial cells. Nanomaterials.

[99] Zhou H, Pandya JK, Tan Y, Liu J, Peng S, Muriel Mundo JL, He L, Xiao H, McClements DJ (2019). Role of mucin in behavior of food-grade TiO2 nanoparticles under simulated Oral conditions. J Agric Food Chem.

[100] Berridge MJ, Bootman MD, Roderick HL (2003). Calcium signalling: dynamics, homeostasis and remodelling. Nat Rev Mol Cell Biol.

[101] Shi H, Magaye R, Castranova V, Zhao J (2013). Titanium dioxide nanoparticles: a review of current toxicological data. Part Fibre Toxicol.

[102] Blevins LK, Crawford RB, Bach A, Rizzo MD, Zhou J, Henriquez JE, Khan DMIO, Sermet S, Arnold LL, Pennington KL, Souza NP, Cohen SM, Kaminski NE (2019). Evaluation of immunologic and intestinal effects in rats administered an E 171-containing diet, a food grade titanium dioxide (TiO2). Food Chem Toxicol.

[103] Evans SM, Ashwood P, Warley A, Berisha F, Thompson RP, Powell JJ (2002). The role of dietary microparticles and calcium in apoptosis and interleukin-1beta release of intestinal macrophages. Gastroenterology.

[104] Nogueira CM, de Azevedo WM, Dagli ML, Toma SH, Leite AZ, Lordello ML, Nishitokukado I, Ortiz-Agostinho CL, Duarte MI, Ferreira MA, Sipahi AM (2012). Titanium dioxide induced inflammation in the small intestine. World J Gastroenterol.

[105] Florence AT (1997). The oral absorption of micro- and nanoparticulates: neither exceptional nor unusual. Pharm Res.

[106] Li H, Zhao X, Ma Y, Zhai G, Li L, Lou H (2009). Enhancement of gastrointestinal absorption of quercetin by solid lipid nanoparticles. J Control Release.

[107] Sheng L, Wang L, Sang X, Zhao X, Hong J, Cheng S, Yu X, Liu D, Xu B, Hu R, Sun Q, Cheng J, Cheng Z, Gui S, Hong F (2014). Nano-sized titanium dioxide-induced splenic toxicity: a biological pathway explored using microarray technology. J Hazard Mater.

[108] Hong J, Wang L, Zhao X, Yu X, Sheng L, Xu B, Liu D, Zhu Y, Long Y, Hong F (2014). Th2 factors may be involved in TiO(2) NP-induced hepatic inflammation. J Agric Food Chem.

[109] Schanen BC, Karakoti AS, Seal S, Drake DR, Warren WL, Self WT (2009). Exposure to titanium dioxide nanomaterials provokes inflammation of an in vitro human immune construct. ACS Nano.

[110] Giovanni M, Yue J, Zhang L, Xie J, Ong CN, Leong DT (2015). Pro-inflammatory responses of RAW264.7 macrophages when treated with ultralow concentrations of silver, titanium dioxide, and zinc oxide nanoparticles. J Hazard Mater.

[111] Lozano-Fernandez T, Ballester-Antxordoki L, Perez-Temprano N, Rojas E, Sanz D, Iglesias-Gaspar M (2014). Potential impact of metal oxide nanoparticles on the immune system: the role of integrins, L-selectin and the chemokine receptor CXCR4. Nanomedicine.

[112] Xue Y, Wu J, Sun J (2012). Four types of inorganic nanoparticles stimulate the inflammatory reaction in brain microglia and damage neurons in vitro. Toxicol Lett.

[113] Sun B, Wang X, Ji Z, Li R, Xia T (2012). NLRP3 inflammasome activation induced by engineered nanomaterials. Small.

[114] Winter M, Beer HD, Hornung V, Kramer U, Schins RP, Forster I (2011). Activation of the inflammasome by amorphous silica and TiO2 nanoparticles in murine dendritic cells. Nanotoxicology.

[115] Baron L, Gombault A, Fanny M, Villeret B, Savigny F, Guillou N, Panek C, le Bert M, Lagente V, Rassendren F, Riteau N, Couillin I (2015). The NLRP3 inflammasome is activated by nanoparticles through ATP, ADP and adenosine. Cell Death Dis.

[116] Romoser AA, Figueroa DE, Sooresh A, Scribner K, Chen PL, Porter W, Criscitiello MF, Sayes CM (2012). Distinct immunomodulatory effects of a panel of nanomaterials in human dermal fibroblasts. Toxicol Lett.

[117] Matricon J, Barnich N, Ardid D (2010). Immunopathogenesis of inflammatory bowel disease. Self Nonself.

[118] Barreau F, Madre C, Meinzer U, Berrebi D, Dussaillant M, Merlin F, Eckmann L, Karin M, Sterkers G, Bonacorsi S, Lesuffleur T, Hugot JP (2010). Nod2 regulates the host response towards microflora by modulating T cell function and epithelial permeability in mouse Peyer's patches. Gut.

[119] Barreau F, Meinzer U, Chareyre F, Berrebi D, Niwa-Kawakita M, Dussaillant M (2007). CARD15/NOD2 is required for Peyer's patches homeostasis in mice. PLoS One.

[120] Lapaquette P, Bringer MA, Darfeuille-Michaud A (2012). Defects in autophagy favour adherent-invasive Escherichia coli persistence within macrophages leading to increased pro-inflammatory response. Cell Microbiol.

[121] Travassos LH, Carneiro LA, Ramjeet M, Hussey S, Kim YG, Magalhaes JG (2010). Nod1 and Nod2 direct autophagy by recruiting ATG16L1 to the plasma membrane at the site of bacterial entry. Nat Immunol.

[122] Abraham C, Cho JH (2009). Inflammatory bowel disease. N Engl J Med.

[123] Powell JJ, Harvey RS, Ashwood P, Wolstencroft R, Gershwin ME, Thompson RP (2000). Immune potentiation of ultrafine dietary particles in normal subjects and patients with inflammatory bowel disease. J Autoimmun.

[124] Hummel TZ, Kindermann A, Stokkers PC, Benninga MA, ten Kate FJ (2014). Exogenous pigment in Peyer patches of children suspected of having IBD. J Pediatr Gastroenterol Nutr.

[125] Ashwood P, Thompson RP, Powell JJ (2007). Fine particles that adsorb lipopolysaccharide via bridging calcium cations may mimic bacterial pathogenicity towards cells. Exp Biol Med.

[126] Ammendolia MG, Iosi F, De Berardis B, Guccione G, Superti F, Conte MP (2014). Listeria monocytogenes behaviour in presence of non-UV-irradiated titanium dioxide nanoparticles. PLoS One.

[127] Moon C, Park HJ, Choi YH, Park EM, Castranova V, Kang JL (2010). Pulmonary inflammation after intraperitoneal administration of ultrafine titanium dioxide (TiO2) at rest or in lungs primed with lipopolysaccharide. J Toxicol Environ Health A.

[128] Baan R, Straif K, Grosse Y, Secretan B, El Ghissassi F, Cogliano V (2006). Carcinogenicity of carbon black, titanium dioxide, and talc. Lancet Oncol.

[129] Proquin H, Jetten MJ, Jonkhout MCM, Garduno-Balderas LG, Briede JJ, de Kok TM (2018). Gene expression profiling in colon of mice exposed to food additive titanium dioxide (E171). Food Chem Toxicol.

[130] Proquin H, Jetten MJ, Jonkhout MCM, Garduno-Balderas LG, Briede JJ, de Kok TM (2018). Transcriptomics analysis reveals new insights in E171-induced molecular alterations in a mouse model of colon cancer. Sci Rep.

[131] Proquin H, Rodriguez-Ibarra C, Moonen CG, Urrutia Ortega IM, Briede JJ, de Kok TM (2017). Titanium dioxide food additive (E171) induces ROS formation and genotoxicity: contribution of micro and nano-sized fractions. Mutagenesis.

[132] Setyawati MI, Sevencan C, Bay BH, Xie J, Zhang Y, Demokritou P (2018). Nano-TiO2 Drives Epithelial-Mesenchymal Transition in Intestinal Epithelial Cancer Cells. Small.

[133] Pinget G, Tan J, Janac B, Kaakoush NO, Angelatos AS, O'Sullivan J, Koay YC, Sierro F, Davis J, Divakarla SK, Khanal D, Moore RJ, Stanley D, Chrzanowski W, Macia L (2019). Impact of the food additive titanium dioxide (E171) on gut microbiota-host interaction. Front Nutr.

[134] Jensen DM, Løhr M, Sheykhzade M, Lykkesfeldt J, Wils RS, Loft S, Møller P (2019). Telomere length and genotoxicity in the lung of rats following intragastric exposure to food-grade titanium dioxide and vegetable carbon particles. Mutagenesis.

[135] Hong F, Wu N, Zhou Y, Ji L, Chen T, Wang L (2017). Gastric toxicity involving alterations of gastritis-related protein expression in mice following long-term exposure to nano TiO2. Food Res Int.

