# Supplementary material for: Titanium dioxide particles from the diet: involvement in the genesis of inflammatory bowel diseases and colorectal cancer
Source: Part Fibre Toxicol. 2021 Jul 30;18:26. doi: 10.1186/s12989-021-00421-2 (PMC8323234; doi:10.1186/s12989-021-00421-2)
Supplement: Supplementary file 1 — Additional file 1. [file 12989_2021_421_MOESM1_ESM.docx]

**Supplementary materials**

Titanium dioxide particles from the diet: involvement in the genesis of inflammatory bowel diseases and colorectal cancer

By Frédérick Barreau^1,2*^, Céline Tisseyre^3^, Audrey Ferrand^1,2^, Marie Carriere^3*^

**Antimicrobial properties of TiO_2_ via photocatalysis.**

TiO_2_-NPs are presently used for their antimicrobial properties, most of the time combined with exposure to UV light. Matsunaga *et al.* conducted the first study by killing *E. coli* bacteria in a short period of time (1–2 h) with TiO_2_-Pt catalysts under UV light [[1](#_ENREF_1)]. Following photocatalytic activation of TiO_2_-NPs, Maness *et al.* have reported that ROS which occurred at the surface of TiO_2_-NPs carried out lipid peroxidation reaction and caused the death of *E. coli* K-12 cells [[2](#_ENREF_2)]. Huang *et al.* reported that photocatalytic reactions increased the permeability of cell membrane and cell death occurred by leaking out of vital components in bacterial cells [[3](#_ENREF_3)]. In addition to their aforementioned photocatalytic properties, owing to the unique properties of their nanosize, TiO_2_-NPs provide more effective elimination of bacteria.

**Analysis of the impact of TiO_2_ on mucus secretion in the lung.**

An abundant literature demonstrates the impact of the TiO_2_-NPs on the mucus secretion from the respiratory system and on the development of the pulmonary diseases like asthma, chronic obstructive pulmonary disease or cystic fibrosis [[4](#_ENREF_4), [5](#_ENREF_5)]. In these pulmonary diseases, mucus hypersecretion and accumulation may lead to recurrent episodes of chronic bacterial infections and chronic inflammatory responses [[5](#_ENREF_5), [6](#_ENREF_6)]. In addition to the impact of TiO_2_-NPs on the induction of inflammatory responses (neutrophil recruitment), epithelial cell death and increased permeability of the pulmonary mucosa is reported [[6](#_ENREF_6), [7](#_ENREF_7)]. TiO_2_-NPs are currently described to also alter directly the secretion of lung mucins. TiO_2_-NPs directly stimulates mucin secretion from human bronchial ChaGo-K1 epithelial cells via a Ca^2+^-mediated signalling pathway [[8](#_ENREF_8)]. *In vivo*, inhalation of TiO_2_-NPs aggravates respiratory symptoms including mucus hypersecretion in patients with chronic airway disease and induced goblet cell hyperplasia associated with an increase of mucin expression like Muc5ac in experimental animal models [[9](#_ENREF_9)].

**Analysis of the impact of TiO_2_ on the immune system after intratracheal administration.**

Intratracheal administration of TiO_2_-NPs in rats results in congestion in the spleen and particulate deposition in the cervical and axillary lymph nodes. Additionally, an increase in both T and B lymphocyte proliferation and enhanced natural killer cell activity is observed [[10](#_ENREF_10)]. Levels of the inflammatory cytokines IL-1 and IL-6, Th1 cytokines (IL-12, TNF-α and IFN-γ) and Th2 cytokines (IL-4, IL-5 and IL-10) are elevated dose-dependently at day 1 and remain elevated for more 14 days after TiO_2_-NPs exposure [[11](#_ENREF_11)].

References

1. Matsunaga T TR, Nakajima T, Wake H. . Photochemical sterilization of microbial cells by semiconductor powders. . FEMS Microbiol Lett. 1985; 29:211–4.

2. Maness PC, Smolinski S, Blake DM, Huang Z, Wolfrum EJ, Jacoby WA. Bactericidal activity of photocatalytic TiO(2) reaction: toward an understanding of its killing mechanism. Appl Environ Microbiol. 1999;65 9:4094-8.

3. Huang Z MP, Blake DM et al. Bactericidal mode of titanium dioxide photocatalysis. J Photochem Photobiol A Chem 2000;130:163-70.

4. Alfaro-Moreno E, Nawrot TS, Nemmar A, Nemery B. Particulate matter in the environment: pulmonary and cardiovascular effects. Curr Opin Pulm Med. 2007;13 2:98-106; doi: 10.1097/MCP.0b013e328013f47e00063198-200703000-00004 [pii].

5. Gwinn MR, Vallyathan V. Nanoparticles: health effects--pros and cons. Environ Health Perspect. 2006;114 12:1818-25.

6. Rogers DF. Physiology of airway mucus secretion and pathophysiology of hypersecretion. Respir Care. 2007;52 9:1134-46; discussion 46-9.

7. Johnston HJ, Hutchison GR, Christensen FM, Peters S, Hankin S, Stone V. Identification of the mechanisms that drive the toxicity of TiO(2 )particulates: the contribution of physicochemical characteristics. Particle and fibre toxicology. 2009;6:33; doi: 1743-8977-6-33 [pii]10.1186/1743-8977-6-33.

8. Chen EY, Garnica M, Wang YC, Chen CS, Chin WC. Mucin secretion induced by titanium dioxide nanoparticles. PLoS One. 2011;6 1:e16198; doi: 10.1371/journal.pone.0016198.

9. Ahn MH, Kang CM, Park CS, Park SJ, Rhim T, Yoon PO, et al. Titanium dioxide particle-induced goblet cell hyperplasia: association with mast cells and IL-13. Respir Res. 2005;6:34; doi: 1465-9921-6-34 [pii]10.1186/1465-9921-6-34.

10. Fu Y, Zhang Y, Chang X, Ma S, Sui J, Yin L, et al. Systemic immune effects of titanium dioxide nanoparticles after repeated intratracheal instillation in rat. Int J Mol Sci. 2014;15 4:6961-73; doi: ijms15046961 [pii]

10.3390/ijms15046961.

11. Park EJ, Yoon J, Choi K, Yi J, Park K. Induction of chronic inflammation in mice treated with titanium dioxide nanoparticles by intratracheal instillation. Toxicology. 2009;260 1-3:37-46; doi: S0300-483X(09)00136-X [pii]10.1016/j.tox.2009.03.005.
